# Geriatric Assessment: ASCO Global Guideline

**DOI:** 10.1200/GO-25-00276

**Published:** 2025-08-27

**Authors:** Cristiane Decat Bergerot, Sarah Temin, Haydee C. Verduzco-Aguirre, Matti S. Aapro, Shabbir M.H. Alibhai, Zeba Aziz, María de la Concepción Pérez de Celis Herrero, Trinanjan Basu, Martine Extermann, Ravindran Kanesvaran, Bogda Koczwara, Kah Poh Loh, Elene Mariamidze, Alex Baleka Mutombo, Vanita Noronha, Grant R. Williams, Enrique Soto-Perez-de-Celis

**Affiliations:** 1 Oncoclinicas&Co - Medica Scientia Innovation Research (MEDSIR), Brasilia, Brazil; 2 American Society of Clinical Oncology (ASCO), Alexandria, VA; 3 Instituto Nacional de Ciencias Médicas y Nutrición, Mexico City, Mexico; 4 Genolier Cancer Center, Genolier, Switzerland; 5 Princess Margaret Cancer Centre, Toronto, ON, Canada; 6 Hameed Latif Hospital, Lahore, Pakistan; 7 Benemérita Universidad Autónoma de Puebla, Puebla, Mexico; 8 HCG Cancer Centre, Mumbai, India; 9 H. Lee Moffitt Cancer Center and Research Institute, Tampa, FL; 10 Division of Medical Oncology, National Cancer Centre, Singapore, Singapore; 11 Department of Medical Oncology Flinders Medical Centre and Flinders University, Adelaide, SA, Australia; 12 University of Rochester Medical Center—Wilmot Cancer Institute, Rochester, NY; 13 Todua Clinic, Tbilisi, Georgia; 14 Kinshasa University Hospital, Kinshasa, Democratic Republic of Congo; 15 TATA Memorial Hospital, Mumbai, India; 16 University of Alabama at Birmingham, Birmingham, AL; 17 University of Colorado Anschutz Medical Campus Aurora, CO

## Abstract

ASCO Guidelines provide recommendations with comprehensive review and analyses of the relevant literature for each recommendation, following the guideline development process as outlined in the ASCO Guidelines Methodology Manual, ASCO Guidelines follow the ASCO Conflict of Interest Policy for Clinical Practice Guidelines.

Clinical Practice Guidelines and other guidance (“Guidance”) provided by ASCO is not a comprehensive or definitive guide to treatment options. It is intended for voluntary use by clinicians and should be used in conjunction with independent professional judgment. Guidance may not be applicable to all patients, interventions, diseases or stages of diseases. Guidance is based on review and analysis of relevant literature, and is not intended as a statement of the standard of care. ASCO does not endorse third-party drugs, devices, services, or therapies and assumes no responsibility for any harm arising from or related to the use of this information. See complete disclaimer in Appendix 1 for more.

**PURPOSE:**

To guide clinicians and policymakers in global resource-constrained settings to assess the geriatric needs of patients older than 65 years with cancer when Maximal-setting guideline–recommended resources are unavailable.

**METHODS:**

A multidisciplinary, multinational Expert Panel reviewed existing ASCO guidelines and conducted modified ADAPTE and formal consensus processes.

**RESULTS:**

An ASCO resource-neutral guideline was adapted for resource-constrained settings, informing one round of formal consensus; recommendations received ≥75% agreement.

**RECOMMENDATIONS:**

The Expert Panel endorses the Maximal-setting guideline’s overarching recommendation that end users utilize geriatric assessment (GA), including essential domains, to identify “vulnerabilities or impairments not routinely captured in oncology assessment for all older patients over 65 years old with cancer.” All care plans for patients with cancer over 65 years old receiving systemic therapy with GA-identified deficits should include GA-guided management. A geriatric evaluation should at a minimum include the use of a brief geriatric screening tool. Tools in the Practical Geriatric Assessment (PGA) are validated in multiple languages, but users may use more appropriate tools in some settings and languages, if they include the relevant guideline-specified domains. Maximal-resource settings of high-income countries have traditionally developed cutoffs for GA-identified deficits, but locally validated research and practice may inform differing cutoffs. If validated all-cause mortality prognosis tools do not adequately represent the setting, clinicians may use actuarial life-expectancy tables with quartiles of overall health status. *Additional information can be found at* www.asco.org/global-guidelines. *It is the view of ASCO that health care clinicians and health care system decision makers should be guided by the recommendations for the highest stratum of resources available. The guideline is intended to complement but not replace local guidelines.*

## Visual Abstract

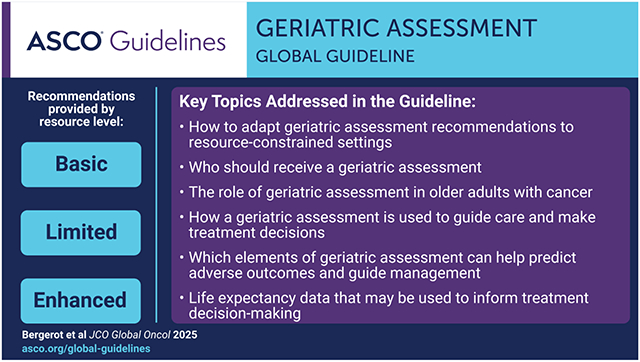

## INTRODUCTION

The purpose of this guideline is to provide expert guidance on cancer-specific geriatric assessment (GA) for older patients in resource-constrained settings (defined in Appendix Table A1) to clinicians (including physicians, nurses, psychologists, nutritionists, physical and occupational therapists), medical care leaders, researchers, cancer advocacy organizations, patients, caregivers, and policymakers. For this guideline, older will be defined as age 65 years and above.^1^ Although the United Nations defines older individuals as those 60 years and older,^2^ other global organizations such as the Organization for Economic Co-operation and Development^3^ or the World Bank^4^ use 65 years as the cutoff for old age. Therefore, and to harmonize the definitions used in this guideline with those of the Maximal-resource setting guideline and with the seminal randomized clinical trials (RCTs) conducted in geriatric oncology, the Panel has decided to use age 65 years for defining an older individual. This guideline’s target population is older adult patients with cancer in resource-constrained settings with limited or no access to clinicians with training in geriatrics and/or to specialists in geriatric oncology.

Currently, over 5 million new cancer cases per year occur in individuals 65 years and older living in low- and middle-income countries (LMICs), representing half of all new cancer cases among older adults globally.^5,6^ By the year 2050, the number of new cancer cases in older adults living in LMICs will more than double, reaching over 12 million cases per year and representing 60% of the global cancer burden among older individuals. Importantly, the most significant increases in incidence will take place in low-income and lower-middle–income countries, with an increase of 177% and 164% in the number of new cancer cases in older adults between 2022 and 2050, respectively.^7^ Similar trends are expected for cancer-related mortality, with 70% of the global cancer-related deaths in older individuals occurring in LMICs by the year 2050. Therefore, there is an enormous need to provide guidance to health care clinicians and policymakers in resource-limited regions of the world regarding the best way to provide high-quality care for the rapidly growing population of older adults with cancer. Existing guidelines may not be directly applicable or feasible in these settings. This guideline is not intended for patients in Maximal-resource settings, as described in Appendix Table A1 (refer to ASCO guidelines originally developed in 2018 and replaced with an update in 2023^8^). It is important to note that the concept of resource limitations is no longer solely applied to countries defined by income according to the World Bank definition; it is a dynamic concept contingent on the stability of the society or region of concern, as well as the concept of human development indices (HDIs). Resource-limited settings may exist within high-income countries (HICs) and vice versa. As stated by Al Sukhun et al,^9^ “The provision of evidence-based and context-appropriate guidance for clinicians has been demonstrated to improve the outcome of patients…supporting a public health approach to best inform clinical decisions in that specific setting.” (p4) In Maximal-resource settings, different standards and resources apply, which may not align with the guidelines provided here. Clinicians in such settings should consider additional resources and approaches applicable to their environment.

The evidence base for any clinical practice recommendations is built on the characteristics of the patients who participated in the underlying studies. In the two largest RCTs of GA and management conducted in the United States supporting the 2023 Maximal-setting guideline that reported race and ethnicity characteristics, 80% of patients were non-Hispanic White, 7% were African American/ Black, and 5%-18% were Hispanic.^10^ Importantly, most RCTs of GA and management have been conducted in resource-rich settings in HICs (although the National Cancer Institute Community Oncology Research Program COACH and GAP studies were conducted in community oncology clinics outside academic medical centers in Maximal settings) and in language-concordant clinician- patient situations, limiting their generalizability and implementation in resource-constrained health care settings.

## GUIDELINE QUESTIONS

This clinical practice guideline addresses four overarching clinical questions posed in the 2023 Maximal-setting guideline update, with the addition of the words “in resource-constrained settings”: (1) What is the role of GA in older adults with cancer to inform specific interventions to improve clinical outcomes in resource-constrained settings? (2) For older patients who are considering undergoing antineoplastic therapy and other systemic treatments, which GA tools and component elements should clinicians use to predict adverse outcomes (including antineoplastic therapy toxicity and mortality) and guide management in resource-constrained settings? (3) What general (ie, noncancer-specific) life expectancy data for community-dwelling patients should clinicians consider to estimate mortality and to best inform treatment decision making for older patients with cancer in resource-constrained settings? (4) How should GA be used to guide management of older patients with cancer in resource-constrained settings?

## METHODS

### Guideline Development Process

This systematic review-based guideline-based and formal consensus guideline product was developed by a multinational, multidisciplinary Expert Panel, which included one patient representative and an ASCO guidelines staff with health research methodology expertise (Appendix Table A2).

The recommendations were informed by the ADAPTE methodology and formal consensus methodology as an alternative to de novo guideline development. The objective of the ADAPTE process is to take advantage of existing guidelines to enhance efficient production, to reduce duplication, and to promote the local uptake of quality guideline recommendations.^11^ ASCO’s adaptation process begins with a systematic review of existing guidelines identified through online searches of PubMed by ASCO guidelines staff to identify candidate guidelines for adaptation. Guidelines are selected for inclusion based on the prespecified criteria. In the case of this guideline, the ASCO Global Guidelines Advisory Group chose to adapt a published Maximal-setting ASCO guideline.

This guideline was partially informed by ASCO’s modified Delphi Formal Expert Consensus methodology,^12^ during which the Expert Panel was supplemented by additional experts recruited to rate their agreement with the drafted recommendations. The entire membership of experts is referred to as the Consensus Panel (Appendix Table A3). In round 1, a total of 26 members (11 of whom were on the Expert Panel) participated. Greater than or equal to 80% of the responses received a rating of either “agree” or “strongly agree” for each of the 16 recommendations. This agreement exceeded the prespecified threshold, and round 2 was not needed.

Three full panel meetings were held, and members were a sked to provide ongoing input on the guideline development protocol, the quality and assessment of the evidence, the generation of recommendations, and draft content and to review and approve drafts during the entire development of the guideline. ASCO staff met routinely with the Expert Panel cochairs and corresponded with the Panel via e-mail to coordinate the process to completion. Ratings for strength of the recommendation and evidence quality are provided with each recommendation, defined in Appendix Table A4. The quality of the evidence for each outcome was assessed using the Cochrane Risk of Bias tool and elements of the GRADE quality assessment and recommendations development process.^13,14^ GRADE quality assessment labels (ie, high, moderate, low, very low) were assigned for each outcome by the project methodologist in collaboration with the Expert Panel cochairs and reviewed by the full Expert Panel. All funding for the administration of the project was provided by ASCO.

### Guideline Review and Approval

The draft recommendations were released to the public for open comment from January 14 through January 28, 2025. Response categories of “Agree as written,” “Agree with suggested modifications,” and “Disagree. See comments” were captured for every proposed recommendation with two written comments received. Of the two respondents, both either agreed with all the recommendations as written or agreed with slight modifications. Expert Panel members reviewed comments from all sources and determined whether to maintain original draft recommendations, revise with minor language changes, or consider major recommendation revisions.

The draft was submitted to three external reviewers with content expertise including from the Cancer and Aging Research Group (CARG) and the International Society of Geriatric Oncology (SIOG). It was rated as high quality, and it was agreed that it would be useful in practice. Review comments such as “important to see literature from the role of GA to improve cancer outcomes conducted in limited-resource settings” and a reminder that in some of the US- and Canadian-based studies reviewed in the Maximal-setting guideline, oncology teams in community oncology clinics with a variable level of multidisciplinary teams available used the interventions were reviewed by the Expert Panel and integrated into the article.

All changes were incorporated into the final manuscript before ASCO Evidence-Based Medicine Committee (EBMC) review and approval. All ASCO global guidelines are ultimately reviewed and approved by the Expert Panel and the ASCO EBMC before submission to the *JCO Global Oncology* for editorial review and consideration for publication.

### Guideline Updating

The ASCO Expert Panel and guidelines staff will work with cochairs to keep abreast of any substantive updates to the guideline. Based on formal review of the emerging literature, ASCO will determine the need to update. The ASCO Guidelines Methodology Manual (available at www.asco.org/guideline-methodology) provides additional information about the guideline update process. This is the most recent information as of the publication date.

## RESULTS

The recommendations were developed through a review of existing Maximal-setting ASCO-published guidelines^8,15^ (2018, 2023) and clinical experience of the panel of experts. The methods of development of each systematic review-based ASCO guideline are available in each publication. A total of two ASCO guidelines on GA were found, and they met criteria for adaptation. The ASCO Expert Panel reviewed these guidelines. The Expert Panel used these guidelines, literature suggested by the Expert Panel, and clinical experience as guides. The Expert Panel acknowledges the effort put forth by the authors and ASCO to produce evidence-based guidelines informing practitioners and institutions who provide care to patients with cancer.

### Literature Search

In addition, a search for new evidence was conducted by ASCO guidelines staff to identify relevant RCTs, systematic reviews, meta-analyses, and guidelines published regarding resource-constrained settings since the 2018 and 2023 guidelines were completed. This evidence was identified through searches of PubMed (2022 January to 2024 June 14 for RCTs; 2022 January to 2024 June 24 for any study design; 2022 January to 2024 June 28 for prognosis of overall cancer mortality) and Google Scholar [2022-2024], including phase III RCTs, some observational studies, and clinical experience. Articles were selected for inclusion in the systematic review based on the following criteria, depending on the Research

Question:

- Population: Older adults with cancer (the definition of older varies between countries and regions); for Question 3: Community-dwelling older adults
- Interventions: GA, for Question 3: geriatric evaluation and management of age-related medical conditions, psychological morbidity, and functional abilities
- Comparisons: Standard or usual care
- Outcomes: Efficacy of GA-informed antineoplastic therapy (including mortality, overall survival), cancer treatment completion, safety, and health-related quality of life (HRQOL)
- Sample size: ≥50 patients across study arms

Publications were excluded from the systematic review if they were (1) meeting abstracts not subsequently published in peer-reviewed journals; (2) pilot studies, editorials, commentaries, letters, news articles, case reports, and narrative reviews; (3) <50 patients across study arms. Please note non-English language publications were included (ASCO resource-neutral systematic reviews usually exclude them).

## SUMMARY OF ADAPTED GUIDELINES

ASCO published its original Maximal-setting guideline in 2018^8^ and updated it in 2023.^15^ The 2018 guideline included four clinical questions, and the 2023 guideline updated its systematic review for two of the four questions (because of new evidence). This globally adapted guideline addresses all four original clinical questions, focusing on their application in resource-constrained settings.

The 2023 guideline update reviewed nine RCTs. The trials were conducted with participants in United States, Canada, France, Denmark, and Australia. The Global Guideline Expert Panel examined the resources required by each of the nine clinical trials (Data Supplement, Table S1). Geriatricians, oncologists, or dually trained clinicians including nurses with or without other team members provided the interventions.

- This global guideline used the same clinical questions as the Maximal-setting guideline (Data Supplement)
- Review and adaptation for resource-constrained settings: The Maximal-setting guideline was developed based on data and/or experience of clinicians and patients in Maximal settings, and therefore, the Panel reviewed and adapted the recommendations for resource-constrained settings on the basis of experience in resource-constrained settings, searched for literature from research conducted in such settings, and then validated the recommendations by formal consensus. In the course of the Panel’s review, the Panel looked at which of the sources and questions for the Practical Geriatric Assessment (PGA) tool, recommended in the Maximal-setting guideline, were translated into the most spoken non-English languages worldwide and identified the specific language (Data Supplement, Table S2).
- The key evidence for the Maximal-setting guidelines included RCTs, systematic reviews, and meta-analyses of RCTs; for a subset of questions, prospective cohort studies (“that evaluated the association of GA-based tools with outcomes of older patients with cancer receiving anti-neoplastic therapy” page 2329, 2018^8^) were also considered. Some recommendations were based on informal consensus.
- Key outcomes and end points in those guidelines included adverse effects or outcomes, overall mortality, overall survival, treatment completion without dose reductions or delays, treatment-related toxicity, patient satisfaction with communication about aging-related concerns, HRQOL, and functional and nutritional status.

## RECOMMENDATIONS

All recommendations are available in Table 1.

### Author’s Note

It is the view of ASCO that health care clinicians and health care system decision makers should be guided by the recommendations for the highest stratum of resources available. The guidelines are intended to complement but not replace local guidelines.

## ROLE OF GA IN IMPROVING CANCER OUTCOMES

In addition to the systematic reviews conducted for the Maximal-setting ASCO guidelines, which this guideline will not rereview, other authors have also reviewed the same clinical trials^20,21^ and the authors of this global guideline would like to refer readers to both these publications.^20,21^ While there is a paucity of literature from LMICs, several groups and institutions have implemented geriatric oncology clinics, mostly in centers of excellence in India, Brazil, and Mexico.^22–26^ The literature review for this guideline found no studies or peer-reviewed publications regarding geriatric oncology prospective research (including implementation outcomes) from resource-constrained settings across other regions of the world, including but not limited to sub-Saharan Africa, other parts of Latin America, Eastern Europe, and Southeast Asia.

### Literature Review and Analysis

The nine RCTs cited in the Maximal-setting guideline were conducted in Maximal settings, including the United States and Canada (GAIN, GAP701, COACH, 5C, HEME RCT),^27–31^ Western Europe,^32–34^ and Australia (INTEGERATE),^35^ including those labeled “community” by Maximal-setting standards. Six of the studies were conducted all or in part in academic centers (GAIN,^27^ GERICO,^32^ 5C,^30^ INTEGE RATE,^35^ EGeSOR,^33^ Ørum^34^). Three studies included Maximal-setting community health care organizations.^29,30,35^ Almost all studies required resources such as geriatricians (GAIN^27^*, GERICO,^32^ 5C,^30^ INTEGERATE,*^35^ EGeSOR,^33^ Ørum,*^34^ HEME RCT^31^; *and/or geriatric-trained clinicians), specialized geriatric oncologists (INTEGERATE,^35^ 5C^30^), and all included multidisciplinary teams. Only GAP70^28^ and COACH^29^ explicitly included patient-reported and/or self-administered GA, and the intervention was delivered by oncology teams in community settings using information obtained from the GA. These resources are currently not often avavailable in most resource-constrained settings.

The Expert Panel reviewed selected nonrandomized studies found in literature searches,^36–40^ and/or suggested studies,^41,42^ conducted in limited-resource settings in Mexico, India, and Brazil.^36–42^ These studies have demonstrated the feasibility of implementing geriatric screening and assessment in resource-limited settings and the possibility of leading to changes in treatment planning and decision making. One of these studies demonstrated that a multidisciplinary team could deliver interventions through telemedicine in a resource-constrained scenario.^38^ The lack of RCTs in resource-constrained settings represents a critical need for future research in geriatric oncology and highlights the need of developing studies looking at implementation across settings, including patient-centered outcomes, process measures, and cost-effectiveness.^36,38–40^

### Clinical Interpretation

Broadly speaking, three geriatric oncology models of care have been studied in RCTs^43^ (1) Specialized Geriatric Oncology Units in academic cancer centers, as in the GAIN RCT^27^; (2) geriatrician-led consultative teams (as in INTEGERATE)^35^; and (3) GA results and recommendations provided to oncologists for decision making (as in GAP- 70).^28^ Some smaller studies conducted in both LMICs and HICs have used telehealth (and in some resource-constrained settings, other personnel may assess patients, eg, nononcologist clinicians, primary care clinicians). Importantly, when conducting a GA, the same essential domains should be evaluated regardless of setting and/or available resources (physical function and performance, functional status, nutrition and weight loss, social support, psychological, comorbidity, cognitive function, GA screening tool, and risk of chemotherapy toxicity).^15^

## SPECIFIC GA TOOLS AND COMPONENT ELEMENTS

During this guideline’s development, the Expert Panel reviewed Table 3 in the Maximal-setting guideline (“Practical Geriatric Assessment Proposed Scoring and Recommendations”) on which domains GA and GA-guided management clinicians should include and modified it for resource-constrained settings. Table 2 in this guideline includes suggestions for each resource stratum. This is based on expert experience. Some of the differences from the Maximal-setting guideline include the addition of suggestions regarding home-based exercise, individualized fall prevention, hydration, nutrient-dense foods, additional nutrition-based interventions, caregiver role, transportation interventions, and psychological services via telehealth.

### Literature Review and Analysis

Because the Maximal-setting guideline included an updated search on GA, this Global guideline’s Expert Panel searched for such publications from research conducted in resource-constrained settings. The study by Verduzco-Aguirre et al,^37^ reviewed in the 2023 Maximal-setting guideline, examined barriers and facilitators among clinicians treating older patients with cancer in Mexico via an observational study including qualitative methods. (Although this study did not meet the study design inclusion criteria, it was used to inform formal consensus.) The study measured the uptake of GA tools among oncologists across various settings in Mexico (including various resource levels). In survey responses, 34% worked at a public institution only and 38% worked in both public and private health care organizations. Only 19% of 196 routinely used GA. The authors also conducted 22 semi-structured interviews. Reported barriers included a lack of geriatricians and clinician knowledge, including of referral processes, and overcrowding in the health care organizations. Facilitators included system-level factors, availability of geriatricians and/or multidisciplinary teams, interest, and patient factors. Study limitations included a low response rate and potential selection bias. The study authors recommend GA training for all health care trainees and medical education interventions. (Based on reviews of other studies on barriers in the Maximal-setting guideline, the ASCO Older Adults Task Force developed the PGA.)

The Maximal-setting guideline recommended the PGA, as does this global guideline, while recognizing that other similar tools may be more appropriate for some settings, cultures, languages, and health care organizations. A summary of the availability of the questions and tools included in the PGA in the most widely spoken languages in the world are included in the Data Supplement (Table S2). This guideline acknowledges that those stakeholders can use such tools if they include the relevant domains mentioned throughout this guideline.

The Panel reviewed selected studies from India, where there may be some patients for which the PGA may not be relevant.

In those studies, most GA tools are similar to those included in studies from the Maximal-setting guideline; however, the investigators used the Hindi Mental Status Examination for patients without literacy. Banerjee et al (two studies and two publications [observational (prospective cohort)]) developed a short GA tool (SCOPE-C) in a tertiary national cancer center for use in India, intend ed for use by nonclinicians.^39,40^ The tool used an interview format as self-report tools were not perceived as feasible since the population was largely of a rural background with limited formal education. The second study developed and validated a screening tool in a prospective cohort of older adults with cancer in India, based on the short GA tool developed by the same authors. The study used the cutoff age of 601 years and domains that are the same or similar to those the PGA uses. The study population was primarily male. Although many of these studies have been conducted in India, similar barriers may be present in other regions of the world that face similar systemic challenges; thus, health care organizations might have to further adapt tools in those settings.

### Clinical Interpretation

No single set of tools is available and has been translated and/or validated in all resource-constrained settings. In settings where the PGA tool may not account for cultural factors, clinicians may use locally developed tools, if they include the essential domains mentioned throughout this guideline.

## USE OF NONCANCER-SPECIFIC LIFE EXPECTANCY DATA

### Literature Review and Analysis

ASCO Maximal-setting guideline Recommendation 3.a. specified the use of the Schonberg or Lee Indices (listed in ePrognosis^19^ as Lee-Schonberg Index). These and other models listed in ePrognosis or in previous ASCO guidelines did not include participants from non-US countries, except for the Suemoto Index. The authors of the Lee-Schonberg Index based it on data from patients from the United States and mostly included non-Hispanic White patients, with an under-representation of older people from diverse racial and/or ethnic backgrounds.^20^ Several of the Maximal- setting–based indices, including those in the study by Lee et al,^45^ cited in the ASCO Maximal-setting guideline have been translated into French, Portuguese, and Spanish, albeit not validated in populations in other countries.^20^ Similarly, the Walter index^46^ was developed among US-based patients 70 years and older who were hospitalized and has been translated into three languages (Data Supplement, Table S3 of ePrognosis languages).

The ASCO 2018 guideline mentioned the study by Suemoto et al,^47^ which this guideline recommends. Briefly, Suemoto published a 10-year mortality prediction index, based on a database of five large cohorts of patients living in the community, largely in the United States and England, who were older than 60 years, and included 13 variables. However, unlike other indices, one of the databases was in Mexico and one was in Brazil. It is important to mention that the Suemoto Index, as well as those recommended in the Maximal-resource guideline, are intended to estimate long-term all cause mortality and therefore may only be applicable for decision making in certain tumor types and stages. ^48^ For other scenarios, disease-specific all-cause mortality prediction tools, or palliative care tools such as the Palliative Performance Scale, also included in ePrognosis, may be useful, though not geriatric-specific. ^20,45,49^

### Clinical Interpretation

In settings represented in the recommended indices, end users should use recommendations in the Maximal-resource guideline. However, since the recommended indices may not apply to all settings around the world, an alternative is using information from life tables, preferably stratified by health status. Several global repositories of life tables are available although the ideal health-adjusted life tables may be better found in local repositories. A comprehensive source for life tables is those issued by the WHO Global Health Observatory.^50^ Another comprehensive repository for global life tables comes from the Source Tools of the Global Burden of Disease study,^51^ which can be searched online (requires registration).

## USE OF GA WITH OLDER PATIENTS WITH CANCER

The Maximal-setting guideline reviewed implementation studies. While there is a paucity of literature in LMICs, several groups and institutions have implemented geriatric oncology clinics, mostly in centers of excellence in India, Brazil, and Mexico.^22–26^ Similar to HICs, there is a lack of information about implementing geriatric oncology in community settings, where resources may be harder to access. An area of research which may be of particular importance for resource-limited settings is the utilization of telemedicine for conducting GAs and for implementing remote interventions.^52^ Initial studies in community settings in the United States and in Brazil have demonstrated the feasibility of this approach, but further research is needed to understand whether remote interventions are equivalent to those delivered in person, as has been done in similar fields like palliative care.^53^

### Literature Review and Analysis

In addition to evidence reviewed in the Maximal-resource setting guidelines, the literature search looked for studies on how to implement GA-guided care. An observational single-arm study evaluated telehealth GA-guided interventions among patients with cancer 65 years and older and eligible for antineoplastic therapy across multiple regions of Brazil.^38^ Of these, 83% was from urban centers, 10% was from remote rural areas, 7% was from low-income tier areas, and 44% was non-White. Patients were remotely referred to a geriatrician, psychologist, and/or nutritionist based on their identified impairments. The results demonstrated a significant improvement in HRQOL and physical function. This study was followed by a RCT conducted among patients 65 years and older, diagnosed with metastatic cancer, and undergoing any line of cancer therapy, regardless of their performance status also in multiple regions of Brazil; 14% was from rural areas.^54^ The results demonstrated significant improvements in physical function, mood, HRQOL, and symptom burden in patients receiving remote GAs and tailored interventions (including psychiatrists, psychologists, nutritionists, exercise physiologists, and pain management specialists). In addition, patients in the intervention arm reported enhanced prognostic awareness over time and greater utilization of active coping strategies.

### Clinical Interpretation

The evidence from the reviewed studies highlights several important clinical insights. In many resource-limited regions of the world, geriatric oncology has already been implemented following various models that are very similar to those used in HICs. Importantly, most of these models have been created in leading institutions, where resources may be more abundant than those institutions across most regions. The observational single-arm study and subsequent RCT by Bergerot et al^54^ demonstrated significant improvements in relevant outcomes using telemedicine, providing evidence toward its use to reach patients in remote rural and low-income areas.

These studies, along with evidence from other disciplines like palliative care, highlight the potential utility of telehealth-based interventions in improving outcomes for older patients with cancer and make it a potentially valuable strategy for optimizing cancer care across varied settings.

## DISCUSSION

In an aging world, the implementation of geriatric oncology is not a luxury but a necessity. Recent research has demonstrated that providing cancer care guided by geriatric principles may lead to better outcomes for older patients with cancer through a reduction in toxicity and an improvement in quality of life. However, most patients do not receive this care because of many factors, including a lack of knowledge and personnel, and institutions in countries with aged societies are struggling to develop systems to provide care for this growing population. For health care systems in resource-constrained regions of the world, where population aging is in an accelerated stage, preparing to provide care for older patients should represent a clinical, research, and policy priority. Through these guideline recommendations, the Expert Panel intends to provide guidance for the development and implementation of geriatric oncology across different resource settings.

Importantly, resource-constrained settings not only exist in LMICs but also in less-developed regions of HICs, particularly in community and/or rural settings, where patients face greater challenges when attempting to access high-quality cancer care than in larger academic institutions in urban centers.^55^ The availability of many critical resources for the management of older adults with cancer, such as geriatric consultation, rehabilitation services, and psychological support, varies widely across community oncology centers, even within the United States.^56^ As such, resource-stratified strategies may also be relevant for many cancer practices within HICs and those in LMICs. In addition, integrating digital health solutions and telemedicine platforms offers a promising avenue for expanding access to geriatric oncology care. These tools have the potential to overcome geographical barriers and workforce shortages, enabling personalized care and follow-up for older adults in remote or underserved areas. Future research should focus on evaluating the effectiveness of these technologies in improving outcomes for older patients with cancer, including their impact on accessibility, quality of care, and patient satisfaction.

## LIMITATIONS OF THE RESEARCH AND FUTURE RESEARCH

There is insufficient evidence from research conducted with populations in resource-constrained settings, including from large swaths of the world, such as sub-Saharan Africa. Literature reviews conducted for this guideline found limited sources to provide new evidence-based recommendations for resource-constrained settings in low- and middle-HDI countries and regions. There is a pressing need to design and conduct studies evaluating the implementation of the various geriatric oncology models across institutions, health care systems, regions, and countries with differing resources. Such studies should not only include geriatric or cancer-specific outcomes but also focus on implementation outcomes, including process measures, workforce utilization, and costs. The Expert Panel encourages researchers, institutions, professional organizations, and funding agencies to work together to rigorously evaluate the implementation of geriatric oncology globally and hopes that this guideline will serve as a blueprint for the design of interdisciplinary programs (including physicians, nurses, and other health care professionals) across various settings. Importantly, implementation science provides a valuable framework for addressing the gaps between evidence and practice in geriatric oncology by identifying barriers and tailoring solutions to specific contexts. Furthermore, global collaboration is essential to advancing geriatric oncology care, particularly in resource-constrained settings.

## MULTIPLE CHRONIC CONDITIONS

Creating evidence-based recommendations to inform treatment of patients with additional chronic conditions, a situation in which the patient might have two or more such conditions—referred to as multiple chronic conditions (MCC)—is challenging. a situation in which the patient might have two or more such (MCC)—is challenging. Patients with MCC are a complex and heterogeneous population, making it difficult to account for all the possible permutations to develop specific recommendations for care. In addition, the best available evidence for treating index conditions, such as cancer, is often from clinical trials whose study selection criteria may exclude these patients to avoid potential interaction effects or confounding of results associated with MCC. As a result, the reliability of outcome data from these studies may be limited, thereby creating constraints for expert groups to make recommendations for care in this heterogeneous patient population.

As many patients for whom guideline recommendations apply present with MCC, any treatment plan needs to consider the complexity and uncertainty created by the presence of MCC and highlights the importance of shared decision making regarding guideline use and implementation. Therefore, in consideration of recommended care for the target index condition, clinicians should review all other chronic conditions present in the patient and take those conditions into account when formulating the treatment and follow-up plan. In addition, the management of MCC in older adults represents an ideal opportunity to interact with other members of the health care team, including geriatricians, primary care clinicians, and other health care professionals providing care for older adults, such as community health care workers, gerontologists, or nurses.

## GUIDELINE IMPLEMENTATION

ASCO guidelines are developed for implementation across health settings. The additional role of Expert Panel members is not only to assess the suitability of the recommendations to implementation in the community setting but also to identify any other barrier to implementation a reader should be aware of. Barriers to implementation include the need to increase awareness of the guideline recommendations among frontline practitioners and patient populations and to provide adequate services in the face of limited resources. The guideline recommendations table and accompanying tools (available at www.asco.org/global-guidelines) were designed to facilitate implementation of recommendations. This guideline will be distributed widely, including through many forms of ASCO communications and the ASCO website.

ASCO believes that cancer clinical trials are vital to inform medical decisions and improve cancer care and that all patients should have the opportunity to participate.

## ADDITIONAL RESOURCES

For current information, including selected updates, supplements, and clinical tools and resources, visit www.asco.org/global-guidelines. The Data Supplement for this includes tables on required resources in studies included in the resource-neutral geriatric oncology guidelines, ePrognosis tools with national origin of population, availability of translated tools, and the search strategy string. Guideline recommendations are also available in the free ASCO Guidelines app (available for download in the Apple App Store and Google Play Store). Listen to key recommendations and insights from panel members on the ASCO Guidelines podcast. The Methodology Manual (available at https://asco.org/guideline-methodology) provides additional information about the ethods used to develop this guideline. Patient information is available at American Cancer Society.^57^

ASCO welcomes your comments on this guideline, including implementation challenges, new evidence, and how this guideline impacts you. To provide feedback, contact us at guidelines@asco.org. Comments may be incorporated into a future guideline update. To submit new evidence or suggest a topic for guideline development, complete the form available at www.asco.org/guidelines.

## INCLUSIVE LANGUAGE

ASCO is committed to promoting the health and well-being of all patients. ASCO guidelines are intended to apply to and be discussed clearly and compassionately with all patients. For this reason, guideline authors use appropriately inclusive language. In instances in which the guideline draws upon data based on research in a specified population (eg, studies on women with ovarian cancer), the guideline authors describe the characteristics and results of the research as reported.

## Supplementary Material

Data Supplement

Summary of Recommendations Table

## Figures and Tables

**Table 1:** Summary of All Recommendations

Clinical Question	Recommendation
*General Note.* The following recommendations (strong or weak) and terminology (Data Supplement) represent reasonable options for patients depending on clinical circumstances and in the context of individual patient preferences. Recommended care should be accessible to patients whenever possible	
1. What is the role of GA in older adults with cancer to inform specific interventions to improve clinical outcomes in resource-constrained settings?	Recommendation 1.1. Basic, Limited, Enhanced: All patients with cancer aged 65 and over receiving systemic therapy and with GA-identified impairments should have GAM included in their care plan. GAM includes using GA results to (1) inform cancer treatment decision-making and (2) address impairments through appropriate interventions, counseling, telemedicine, and/or referrals. (Evidence quality: Moderate-High; Strength of recommendation: Strong)
2. For older patients who are considering undergoing antineoplastic therapy and other systemic treatments, which GA tools and component elements should clinicians use to predict adverse outcomes (including antineoplastic therapy toxicity and mortality) and guide management in resource-constrained settings?	Recommendation 2.1. Basic: A geriatric evaluation should include at a minimum the use of a brief geriatric screening tool (G8 tool with a cutoff of ≤14 points is recommended). (Evidence quality: Moderate; Strength of recommendation: Strong) Recommendation 2.1. Limited, Enhanced: A GA should include high-priority aging-related domains known to be associated with outcomes in older patients with cancer to include assessment of physical and cognitive function, emotional health, comorbid conditions, polypharmacy, nutrition, and social support. (Evidence quality: High; Strength of recommendation: Strong)
	Recommendation 2.2. Basic: For patients who are identified as potentially vulnerable using a screening tool, the Panel recommends the PGA as one option for conducting a GA. See the PGA tool [https://cdn.bfldr.com/KOIHB2Q3/as/fr5tbfs65q37wzkqhnn3fn7/2023Practical-Geriatric-Assessment]^16^ and associated videos (How to Do A GA [https://youtu.be/jnaQIjOz2Dw],^17^ What to do with the Results of a GA [https://youtu.be/nZXtwaGh0Z0]^18^). (Evidence quality: Moderate; Strength of recommendation: Weak) Recommendation 2.2. Limited and Enhanced: The Panel recommends the PGA as one option for this purpose. See the PGA tool [https://cdn.bfldr.com/KOIHB2Q3/as/fr5tbfs65q37wzkqhnn3fn7/2023Practical-Geriatric-Assessment]^16^ and associated videos (How to Do a GA [https://youtu.be/jnaQIjOz2Dw],^17^ What to do with the Results of a GA [https://youtu.be/nZXtwaGh0Z0]^18^). (Evidence quality: Moderate; Strength of recommendation: Weak)
	Recommendation 2.3. (all levels): Although the tools available in the PGA are available and validated in multiple languages, other similar tools may be more appropriate for some settings and languages, and those could be used if they include the relevant domains mentioned in Recommendation 2.1. (Evidence quality: Moderate; Strength of recommendation: Strong)
	Recommendation 2.4. (all levels): Cutoffs for GA-identified impairments have traditionally been developed in Maximal-resource settings of high-income countries. Therefore, differing cutoffs may be informed by locally validated research and practice. (Evidence quality: Moderate; Strength of recommendation: Weak)
3. What general (ie, noncancer-specific) life expectancy data for community-dwelling patients should clinicians consider for estimating mortality and best inform treatment decision making for older patients with cancer in resource-constrained settings?	Recommendation 3.1. (all levels): In settings not adequately represented in the validated tools listed at ePrognosis,^19^ clinicians may use actuarial life-expectancy tables, with preference given to those that consider quartiles of overall health status. (Evidence quality: Moderate; Strength of recommendation: Weak)
	Recommendation 3.2. (all levels): Based on the best clinical opinion of the Expert Panel, clinicians should use one of the validated tools listed at ePrognosis to estimate life expectancy ≥4 years. a. For settings outside the United States and Canada, the Expert Panel especially recommends the Suemoto Index. The most common variables considered in these indices include age, sex, comorbidities (eg, diabetes, COPD), functional status (eg, ADLs, IADLs, mobility), health behaviors and lifestyle factors (eg, smoking status, body mass index), and self-rated health b. Several indices have “presence of cancer” as a relevant variable, answering “no” to this question will allow for noncancer life expectancy, to consider competing risks of mortality. (Evidence quality: Moderate; Strength of recommendation: Strong that it predicts mortality; Weak that it improves outcomes or improves decision making)
4. How should GA be used to guide management of older patients with cancer in resource-constrained settings?	Recommendation 4.1. Basic: Delphi consensus panels of experts have established approaches for implementing GA-guided care processes in older adults with cancer. The Expert Panel recommends that clinicians apply the results of GA to develop an integrated and individualized plan for patients that informs treatment selection by helping to estimate risks for adverse outcomes and to identify nononcologic problems that may be amenable to intervention. Based on clinical experience and the results of formal expert consensus studies, the Expert Panel suggests that clinicians consider G8 screening tool plus or minus GA results and when recommending treatment and that the information be provided to patients and caregivers to guide decision making for treatment. In addition, clinicians should implement targeted, GA-guided interventions to manage nononcologic problems (Table 2) *Qualifying statement for Recommendation 4.1. Basic:* Most of these processes have been developed in Maximal-resource settings. Therefore, the implementation of GA-guided care may need to be adapted to the local context following the interventions listed in Table 2. A model of care utilizing the GA to inform clinicians to adapt treatment decisions for older patients with cancer may be reasonable in Basic settings Recommendation 4.1. Limited: Delphi consensus panels of experts have established approaches for implementing GA-guided care processes in older adults with cancer. The Expert Panel recommends that clinicians apply the results of GA to develop an integrated and individualized plan for patients that informs treatment selection by helping to estimate risks for adverse outcomes and to identify nononcologic problems that may be amenable to intervention. Based on clinical experience and the results of formal expert consensus studies, the Expert Panel suggests that clinicians consider GA results when recommending treatment and that the information be provided to patients and caregivers to guide decision making for treatment. In addition, clinicians should implement targeted, GA-guided interventions to manage nononcologic problems (Table 2) *Qualifying statement for Recommendation 4.1. Limited:* Most of these processes have been developed in Maximal- or Enhanced-resource settings. Therefore, the implementation of GA-guided care may need to be adapted to the local context following the interventions listed in Table 2. A model of care which uses the GA to inform oncologists about adapting treatment decisions, along with appropriate referrals and comanagement for older patients with cancer may be reasonable in Limited settings Recommendation 4.1. Enhanced: Delphi consensus panels of experts have established approaches for implementing GA-guided care processes in older adults with cancer. The Expert Panel recommends that clinicians apply the results of GA to develop an integrated and individualized plan for patients that informs treatment selection by helping to estimate risks for adverse outcomes and to identify nononcologic problems that may be amenable to intervention. Based on clinical experience and the results of formal expert consensus studies, the Expert Panel suggests that clinicians consider GA results when recommending treatment and that the information be provided to patients and caregivers to guide decision making for treatment. In addition, clinicians should implement targeted, GA-guided interventions to manage nononcologic problems (Table 2) Rating for all settings: (Evidence quality: Moderate; Strength of recommendation: Weak)

NOTE. The strength of the recommendation is defined as follows: Strong: In recommendations for an intervention, the desirable effects of an intervention outweigh its undesirable effects. In recommendations against an intervention, the undesirable effects of an intervention outweigh its desirable effects. All or almost all informed people would make the recommended choice for or against an intervention. Conditional/Weak: In recommendations for an intervention, the desirable effects probably outweigh the undesirable effects, but appreciable uncertainty exists. In recommendations against an intervention, the undesirable effects probably outweigh the desirable effects, but appreciable uncertainty exists. Most informed people would choose the recommended course of action, but a substantial number would not.

Abbreviations: ADLs, activities of daily living; GA, geriatric assessment; GAM, GA-guided management; IADLs, instrumental activities of daily living; PGA, practical geriatric assessment.

**Table 2:** Adaptation of Table 3 in Maximal-Setting Guideline

Deficit	Basic	Limited (to add to Basic)	Enhanced (to add to Limited plus Basic)
Physical function/falls	Check orthostatic blood pressure and adjust medications Provide fall prevention and general physical activity/exercise handouts Weigh risk and benefits of cancer treatment options incorporating information about physical performance Encourage home-based exercises to improve strength and stability	Consider outpatient physical/occupational therapy	Consider home-based physical therapy Request gait/assistive device evaluation, lower extremity strength, and balance training Referral for home safety evaluation
Functional status	Consider treatment modifications, particularly in the noncurative treatment setting: single agent rather than doublet, modified (reduced) dosage (dose de-escalation), treatment schedule modification Consider more frequent toxicity checks Encourage regular physical activity (eg, walking, light stretching)	Consider outpatient physical therapy Provide guidance on safe exercise routines to maintain mobility and independence	Consider home-based physical therapy Request gait/assistive device evaluation, lower extremity strength, and balance training Consider occupational therapy
Nutrition/weight loss	Discuss concerns related to nutrition and how potential treatment may affect nutrition Consider recommendations and/or handouts for nutritional supplements, liberalize calorie restricted diets, small frequent meals and/or high protein snacks Use caution with highly emetogenic regimens Use aggressive antiemetic therapy Education on nausea and vomiting Educate on hydration importance and strategies to maintain fluid intake Encourage weight monitoring to track unintentional weight loss	Referral to a nutritionist/dietitian Referral to a dentist (for oral health issues affecting nutrition, eg, mucositis) Referral to physical therapy Offer guidance on easy-to-prepare, nutrient-dense foods suitable for patients with limited energy	Referral to speech therapy for issues affecting chewing or swallowing Meals-on-wheels or social work for food assistance at home Referral to occupational therapy Consider medications for loss of appetite
Social support	Discuss the availability of social support at home Discuss who to contact in case of emergency Confirm documented health care proxy Identify primary caregiver or family member responsible for daily support Provide education on patient and caregiver rights (eg, advanced directives) Review available community supports such as peer support groups and faith-based organizations	Referral to social work Facilitate connection to local or remote support resources for patients and caregivers Assist with transportation needs	Visiting community nursing services In-person lifeline emergency services
Depression/anxiety	Review history of psychiatric disorders Initiate pharmacologic therapy Assess suicide risk or elder abuse Provide emotional support through family/caregiver involvement Link to community resources (including spiritual)	Referral to a psychologist Referral to a social worker Referral to psychiatry Referral to palliative care	Referral to a psycho-oncologist Referral to health care organization-provided spiritual counseling/chaplaincy Integrated care plans tailored to comorbid conditions and cancer treatment
Comorbidity	Discuss how comorbidities affect risks and benefits of treatment choices Initiate direct communication and management with primary care clinicians about comorbidities and medication Modify schedule if there are concerns about tolerability If history of diabetes, avoid neurotoxic agents (use steroids with caution) If history of heart disease, consider minimizing volume of agents If history of liver or kidney disease, adjust medication as appropriate Ensure wearing glasses Review the medication list	Referral for hearing aid evaluation, if clinically indicated Referral for a vision specialist, if clinically indicated Review medication list, referral to another clinician (geriatrician), assess adherence and minimize medications as much as possible	Test for glaucoma, if clinically indicated Consider involving a clinical pharmacist
Cognition	Provide explicit and written instruction for appointments, medications, and treatments Identify the primary caregiver or family member responsible for daily support and elicit input from caregivers regarding the patient’s cognition Assess decision-making capacity Assess for potential elder abuse	Referral to a geriatrician or neurologist for follow-up Referral to occupational therapy referral	Referral to a cognitive specialist Neuropsychological testing
Abnormal geriatric screening	Consider using the CARG chemotherapy toxicity^2^ calculator to assess the risk of toxicity and guide decision-making Considering administering a GA	Administer a GA	Administer a GA
Intermediate or high risk of chemotherapy toxicity according to CARG score	Consider administering a GA Consider treatment modifications, particularly in the noncurative treatment setting: single agent rather than doublet, modified dosage, treatment schedule modification Consider more frequent toxicity checks	Administer a GA	Administer a GA

NOTE. 1. Table adapted from the study by Dale et al 2023,^15^ 2. Chemo-Toxicity Calculator—Cancer and Aging Research Group.^44^

Abbreviations: CARG, Cancer and Aging Research Group; GA, geriatric assessment.

## TARGET POPULATION AND AUDIENCE

### Target Population

People with cancer who are 65 years and older in resource-constrained regions or countries and/or marginalized populations in three levels of resource settings.

### Target Audience

Clinicians (including physicians, nurses, psychologists, nutritionists, physical and occupational therapists), medical care leaders, patients, caregivers, and policy- makers in resource-constrained settings.

## RELATED ASCO GUIDELINES

- Palliative Care for Patients With Cancer^58^ (https://ascopubs.org/doi/10.1200/JCO.24.00542)
- Patient-Clinician Communication^59^ (http://ascopubs.org/doi/10.1200/JCO.2017.75.2311)
- Palliative Care in the Global Setting^60^ (https://ascopubs.org/doi/10.1200/JGO.18.00026)
- Practical Assessment and Management of Vulnerabilities in Older Patients Receiving Systemic Cancer Therapy^15^ (https://ascopubs.org/doi/10.1200/JCO.23.00933)

## APPENDIX 1. GUIDELINE DISCLAIMER

The Clinical Practice Guidelines and other guidance published herein are provided by ASCO to assist clinicians in clinical decision making. The information herein should not be relied upon as being complete or accurate, nor should it be considered as inclusive of all proper treatments or methods of care or as a statement of the standard of care. With the rapid development of scientific knowledge, new evidence may emerge between the time information is developed and when it is published or read. The information is not continually updated and may not reflect the most recent evidence. The information addresses only the topics specifically identified therein and is not applicable to other interventions, diseases, or stages of diseases. This information does not mandate any particular course of medical care. Further, the information is not intended to substitute for the independent professional judgment of the treating clinician, as the information does not account for individual variation among patients. Recommendations specify the level of confidence that the recommendation reflects the net effect of a given course of action. The use of words like “must,” “must not,” “should,” and “should not” indicates that a course of action is recommended or not recommended for either most or many patients, but there is latitude for the treating physician to select other courses of action in individual cases. In all cases, the selected course of action should be considered by the treating clinician in the context of treating the individual patient. Use of the information is voluntary. ASCO does not endorse third party drugs, devices, services, or therapies used to diagnose, treat, monitor, manage, or alleviate health conditions. Any use of a brand or trade name is for identification purposes only. ASCO provides this information on an “as is” basis and makes no warranty, express or implied, regarding the information. ASCO specifically disclaims any warranties of merchantability or fitness for a particular use or purpose. ASCO assumes no responsibility for any injury or damage to persons or property arising out of or related to any use of this information, or for any errors or omissions.

## APPENDIX 2. GUIDELINE AND CONFLICTS OF INTEREST

The Expert Panel was assembled in accordance with ASCO’s Conflict of Interest Policy Implementation for Clinical Practice Guidelines (“Policy,” found at http://www.asco.org/guideline-methodology). All members of the Expert Panel completed ASCO’s disclosure form, which requires disclosure of financial and other interests, including relationships with commercial entities that are reasonably likely to experience direct regulatory or commercial impact as a result of promulgation of the guideline. Categories for disclosure include employment; leadership; stock or other ownership; honoraria, consulting or advisory role; speaker’s bureau; research funding; patents, royalties, other intellectual property; expert testimony; travel, accommodations, expenses; and other relationships. In accordance with the Policy, the majority of the members of the Expert Panel did not disclose any relationships constituting a conflict under the Policy.

**Table A1:** Framework of Resource Stratification

Setting
Basic
Core resources or fundamental services that are absolutely necessary for any public health/primary health care system to function; basic-level services typically are applied in a single clinical interaction. Vaccination is feasible for highest-need populations
Limited
Second-tier resources or services that are intended to produce major improvements in outcomes such as incidence and cost-effectiveness and are attainable with limited financial means and modest infrastructure; limited-level services may involve single or multiple interactions. Universal public health interventions feasible for a greater percentage of population than the primary target group
Enhanced
Third-tier resources or services that are optional but important; enhanced-level resources should produce further improvements in outcome and increase the number and quality of options and individual choice (perhaps ability to track patients and links to registries)
Maximal
May use high-resource settings’ guidelines
High-level/state-of-the-art resources or services that may be used/available in some high-resource countries and/or may be recommended by high-resource setting guidelines that do not adapt to resource constraints, but that nonetheless should be considered a lower priority than those resources or services listed in the other categories on the basis of extreme cost and/or impracticality for broad use in a resource-limited environment

NOTE. Data adapted from the studies by Anderson and Horton et al.^61,62^ To be useful, Maximal-level resources typically depend on the existence and functionality of all lower-level resources.

**Table A2:** Global Geriatric Assessment Guideline Expert Panel Membership

Name	Affiliation	Role or Area of Expertise
Cristiane Decat Bergerot, PhD, BS, MS (Cochair)	Oncoclinicas & Co—Medica Scientia Innovation Research (MEDSIR), Brasilia, Brazil	Clinical Psychologist; Psycho-Oncology
Enrique Soto-Perez-de-Celis, MD, PhD, MSc, MS (Co-Chair)	University of Colorado Anschutz Medical Campus, Aurora, CO	Geriatric Oncology
Matti S. Aapro, MD	Genolier Cancer Center, Genolier, Nyon, Switzerland	Medical Oncology
Shabbir M.H. Alibhai, MD, MSc	Princess Margaret Cancer Centre, Toronto, ON, Canada	Geriatrics
Zeba Aziz, MD	Hameed Latif Hospital, Lahore, Pakistan	Medical Oncology
Alex Mutombo Baleka, MD, PhD	Kinshasa University Hospital, Kinshasa, Democratic Republic of Congo	Obstetrics/Gynecology
Trinanjan Basu, MD	HCG Cancer Centre, Mumbai, India	Radiation Oncology
Martine Extermann, MD, PhD	H. Lee Moffitt Cancer Center and Research Institute, Tampa, FL	Geriatric Oncology
Ravindran Kanesvaran, MD	National Cancer Centre, Singapore, Singapore	Geriatric Oncology
Bogda Koczwara, BMBS	Flinders Medical Centre and Flinders University, Adelaide, SA, Australia	Medical Oncology Implementation Science Cancer Survivorship
Kah Poh Loh, MBBCh BAO, MS	University of Rochester Medical Center—Wilmot Cancer Institute, Rochester, NY	Geriatric Hematology/Oncology
Elene Mariamidze, MD	Todua Clinic, Tbilisi, Georgia	Medical Oncology
Vanita Noronha, MD	TATA Memorial Hospital, Mumbai, India	Medical Oncology
María de la Concepción Pérez de Celis Herrero, MSc, PhD	Benemérita Universidad Autónoma de Puebla, Puebla, Mexico	Advocate
Haydee C. Verduzco-Aguirre, MD	Instituto Nacional de Ciencias Médicas y Nutrición, Mexico City, Mexico	Medical Oncology, Medical Education
Grant R. Williams, MD, MSPH	University of Alabama at Birmingham, Birmingham, AL	Medical Oncology
Sarah Temin, MSPH	ASCO, Alexandria, VA	ASCO Guideline Staff (Health Research Methods)

**Table A3:** Global Geriatric Assessment Guideline Consensus Panel Membership

Name	Affiliation
Thierry Alcindor, MD	Dana-Farber Cancer Institute, Boston, MA
Daniel A. Anaya, MD	H. Lee Moffitt Cancer Center and Research Institute, Tampa, FL
Alexandra Mendes Barreto Arantes, MD	Instituto OncoVida, Brasília, Brasil
Mehmet Artac, MD, PhD	Necmettin Erbakan University Meram Medical Faculty Medical Oncology Department, Konya, Turkey
Pauline Anne Peronilla Cauton, MD	Cardinal Santos Medical Center, San Juan City, Philippines
Onyema Chido-Amajuoyi, MD, MPH	University of Washington/Fred Hutchinson Cancer Center Seattle, WA
Raul Cordoba, MD, PhD, MS	Fundacion Jimenez Diaz University Hospital, Madrid, Spain
Henry Leonidas Gomez, MD, PhD	Oncosalud-AUNA, Santiago de Surco, Peru
Adeline Cruz Gonzales, MD	De La Salle University Medical Center, San Pedro, Laguna, Philippines
Gustavo Gössling, MD	Latin American Cooperative Oncology Group, LACOG, Porto Alegre, Brasil
Coumba Gueye, MD	Cancer Inst of Dakar—Aristide Le Dantec Hospital, Plateau-Dakar, Dakar, Senegal
Muhammad Rafiqul Islam, MD, MBBS, MPH, FACP	National Institute of Cancer and Research Hospital, Dhaka, Bangladesh
Adedayo Joseph, MD, MBBS	NSIA-LUTH Cancer Center, Lagos University Teaching Hospital, Lagos, Nigeria
Diah Martina, MD	Division of Hematology-Medical Oncology Department of Internal Medicine Dr Cipto Mangunkusumo General Hospital/Faculty of Medicine Universitas Indonesia, Jakarta, Indonesia
Paola Catherine Montenegro, MD	AUNA, Miraflores, Pero
Aylen Vanessa Ospina Serrano, MD, MSc	Hospital Universitario Puerta De Hierro, Madrid, Spain
Purvish Mahendra Parikh, MD, PhD, MBA, MNAMS	Mahatma Gandhi University of Medical Sciences and Technology, Mumbai, India
Sophie Pilleron, PhD	Luxembourg Institute of Health, Strassen, Luxembourg
Arun Shahi, MD, MPH	Patan Academy of Health Sciences, Kathmandu, Nepal
Nisha Mohd Shariff, MBBCh	University Malaya Medical Center, Selangor, Malaysia
Maria del Rosario Sifon, MD	CEMIC, Ciudad Autónoma de Buenos Aires, Argentina
Gilliosa Spurrier-Bernard, BSc, MSc	MELANOMEFRANCE and MPNE, Teilhet, France
Kaori Tane, MD	Hyogo Cancer Center, Akashi-Shi, Japan
Piotr Jan Wysocki, MD, PhD	Jagiellonian University—Medical College Hospital, Kraków, Poland

NOTE. Disclosures of potential conflicts of interest provided by the consensus panel members are available in the guideline supplement.

**Table A4:** Recommendation Rating Definitions

Term	Definitions
Evidence quality	
High	We are very confident that the true effect lies close to that of the estimate of the effect
Moderate	We are moderately confident in the effect estimate: The true effect is likely to be close to the estimate of the effect, but there is a possibility that it is substantially different
Low	Our confidence in the effect estimate is limited: The true effect may be substantially different from the estimate of the effect
Very Low	We have very little confidence in the effect estimate: The true effect is likely to be substantially different from the estimate of effect
Strength of recommendation	
Strong	In recommendations for an intervention, the desirable effects of an intervention outweigh its undesirable effects In recommendations against an intervention, the undesirable effects of an intervention outweigh its desirable effects All or almost all informed people would make the recommended choice for or against an intervention
Conditional/Weak	In recommendations for an intervention, the desirable effects probably outweigh the undesirable effects, but appreciable uncertainty exists In recommendations against an intervention, the undesirable effects probably outweigh the desirable effects, but appreciable uncertainty exists Most informed people would choose the recommended course of action, but a substantial number would not

NOTE. GRADE Handbook, Schünemann et al.^63,64^
