# Supplementary material for: Geriatric Assessment: ASCO Global Guideline
Source: JCO Glob Oncol. Author manuscript; Available in PMC 2026 Apr 1. (PMC13039670; doi:10.1200/GO-25-00276)
Supplement: Data Supplement [file NIHMS2154859-supplement-Data_Supplement.pdf]

ASCO Guidelines

Geriatric Assessment: ASCO Global Guideline

Supplement

Table of Contents

Data Supplement 1: Additional Evidence Tables 2
Table 1. Studies included in general geriatric oncology guidelines with required resources 2
Table 2. Availability of translated tools (sources of Practical Geriatric Assessment) 5
Table 3. ePrognosis tools with national origin of population 15
Data Supplement 2: Search Strategy String and Dates 20
Data Supplement 3: Search Yields 23
Data Supplement 4: Clinical Questions 23
Data Supplement 5: Development of Recommendations Using Formal Consensus 24
Data Supplement 7: GLIDES Action Verb Glossary 30
Data Supplement 8: What is Geriatric Assessment 32
References 36

Data Supplement 1: Additional Evidence Tables

Table 1. Studies included in general geriatric oncology guidelines with required resources

**Author/Study/ Design**	**Setting**	**Intervention**	**Required resources**
Li<br>GAIN [1a]<br>RCT<br>*	Academic, NCI-designated cancer center<br>Age 65+<br>US	Intervention and referrals based on GA and Fulmer SPICES<br>Follow-up by NP (patient education, care coordination, additional specialty referrals)	Geriatrics-trained MDT (oncologist, NP, pharmacist, physical therapist, occupational therapist, social worker, nutritionist) in same center
Mohile 2021<br>GAP70+ [2a]<br>RCT<br>*	Community oncology practices affiliated with NCORP<br>Age 70+<br>US	GA summary and management recommendations, including cancer treatment considerations sent to the oncologist	GA mostly patient reported, completed at home or in clinic<br>Objective cognition and physical performance assessments administered by practice staff/research coordinators<br>Tailored GAM recommendations generated using a web-based platform<br>Brief GAM training for oncologists<br>Capacity for referrals
Lund<br>GERICO[3a]<br>RCT<br>*	Academic<br>Age 70+<br>Denmark	CGA at the start of chemotherapy: medication review, assessment of comorbidity, psycho-cognitive function, nutritional, functional and physical status<br>Appropriate interventions	Geriatrician<br>Dietitian<br>Physical exercise programme<br>Capacity for referrals
Mohile 2020<br>COACH[4a]<br>RCT<br>*	Community oncology practices affiliated with NCORP<br>Age 70+<br>US	GA (functional status, physical performance, comorbidity, polypharmacy, cognition, nutrition, psychological health, social support)	GA mostly patient-reported<br>Trained coordinators completed objective performance and cognitive measures<br>GA scores entered into a web-based folder that created a tailored GA

Author/Study/ Design	Setting	Intervention	Required resources
			summary and guided recommendations<br>Brief GA training for oncologists<br>Capacity for referrals
Puts 2023<br>5C[5a]<br>RCT<br>*	Academic, tertiary cancer centers<br>Age 70+<br>Canada	CGA and management<br>Cognition, mood, medication use, nutritional status, comorbidities, functional status, fall risk, sensory function, social Support	Gerionc team: RN and geriatrician (geriatrician available by telehealth in 2/7 sites)<br>Capacity for referrals
Soo<br>INTEGERATE[6a]<br>RCT<br>*	Academic, two metropolitan teaching hospital, one metropolitan hospital (same public system)<br>Age 70+<br>Australia	CGA: comorbidities, medication review, functioning (physical, cognitive, psychological and social), frailty, falls, nutrition, sensory impairment, immunization status, advanced care planning and chemotherapy toxicity risk<br>Personalized management plan	Dual-trained geriatric oncologist (role as geriatrician embedded within the oncology service)<br>Capacity for referrals
**Study/Design**	Setting	Intervention	Required resources
Paillaud<br>EGeSOR[7a]<br>RCT<br>*	13 centers (10 academic, 3 non-academic)<br>Age 65+<br>France	GA-driven intervention and follow-up<br>Intervention program based on problems detected in GA-related domains, 5 comorbidities (AF, CHF, diabetes, coronary artery disease, hypertension), medication review, patient education for self-management of comorbidities, and information on cancer treatments	Geriatrician<br>Participation of geriatrician in MDT<br>Nurse (“if necessary”)<br>Follow-up by geriatrician (7 visits over the course of treatment)<br>Capacity for referrals

Author/Study/ Design	Setting	Intervention	Required resources
Ørum<br>Tailored GA follow-up[8a]<br>RCT	Academic, single center<br>Age 70+<br>Denmark	CGA (polypharmacy, ADL/IADL, comorbidity, cognition, mood, nutrition)<br>Interventions (pharmacological, physical, nutritional, social)	Geriatric MDT: geriatrician and trained geriatric nurse<br>Tailored follow-up to be done within 90 days, in hospital (outpatient or inpatient), in the patient's own home or by phone<br>Capacity for referrals
Dumontier<br>HEME RCT[9a]<br>RCT<br>*	Academic, NCI-designated cancer center<br>Age 75+<br>US	Geriatric consultation	Geriatrician embedded in oncology clinic<br>Capacity for referrals in same center

Notes: [a] Included in 2023 parent guidelines

Abbreviations. ADL, activities of daily living; AF, atrial fibrillation; CGA, comprehensive geriatric assessment; CHF, chronic heart failure; GA, geriatric assessment; GAM, GA-guided management; IADL, instrumental activities of daily living; MDT, multidisciplinary team; NCI, National Cancer Institute; NCORP, NCI Community Oncology Research Program; NP, nurse practitioner; RCT, randomized controlled trial; RN, registered nurse; US, United States

Table 2. Availability of translated tools (sources of Practical Geriatric Assessment)

#	Description	English language source	Chinese	Spanish	Hindi	Portuguese	Arabic	French
1	How many times have you fallen in the last 6 months?	Direct question (self-description of falls)	Y	Y	Y	Y	Y	Y
2	Does your health limit you in walking one block?	OARS Multidimensional Functional Assessment	Chiu HC, Chen YC, Mau LW, et al. An evaluation of the reliability and validity of the Chinese-version OARS Multidimensional Functional Assessment Questionnaire. Chinese J Public Health. 1997;16 :119–132.	https://www.elsevier.es/es-revista-atencion-primaria-27-articulo-version-espanola-del-oars-multidimensional-14256	NA	https://cdn.publisher.gn1.link/ggaging.com/pdf/v2n4a02.pdf Characteristics of instruments of functional assessment in the elderly persons living within the community in Brazil Características de instrumentos de avaliação funcional em idosos residentes na comunidade no Brasil	Assessment of the functional status of elderly subjects in Qassim Region, Saudi Arabia Mohammed A. Al-Mahadi, FFCM KFU, and Ahmed G. Elzubier, MRCP. View all authors and affiliations Volume 117, Issue 5 https://doi.org/10.1177/146642409711700509	Ferring D, Windle G, Heiss C, et al. (2001). European Study of Adult Well-Being (ESAW): Comparative report on physical health and functional status. EU 5th Framework Project, Contract number: QLK6-CT-2001-00280
3	Does your health now limit you in climbing one flight of stairs?	OARS Multidimensional Functional Assessment	Chiu HC, Chen YC, Mau LW, et al. An evaluation of the reliability and validity of the Chinese-version OARS Multidimensional Functional Assessment Questionnaire. Chinese J Public	https://www.elsevier.es/es-revista-atencion-primaria-27-articulo-version-espanola-del-oars-multidimensional-14256	NA	https://cdn.publisher.gn1.link/ggaging.com/pdf/v2n4a02.pdf Characteristics of instruments of functional assessment in the elderly persons living within the community in Brazil Características de instrumentos de	Assessment of the functional status of elderly subjects in Qassim Region, Saudi Arabia Mohammed A. Al-Mahadi, FFCM KFU, and Ahmed G. Elzubier, MRCP. View all authors and affiliations Volume 117, Issue 5 https://doi.org/10.1177/146642409711700509	Ferring D, Windle G, Heiss C, et al. (2001). European Study of Adult Well-Being (ESAW): Comparative report on physical health and functional status. EU 5th Framework Project, Contract number: QLK6-CT-2001-00280

#	Description	English language source	Chinese	Spanish	Hindi	Portuguese	Arabic	French
			Health. 1997;16 :119–132.			avaliação funcional em idosos residentes na comunidade no Brasil		
4	Can you get to places out of walking distance…	OARS Multidimensional Functional Assessment	Chiu HC, Chen YC, Mau LW, et al. An evaluation of the reliability and validity of the Chinese-version OARS Multidimensional Functional Assessment Questionnaire. Chinese J Public Health. 1997;16 :119–132.	https://www.elsevier.es/es-revista-atencion-primaria-27-articulo-version-espanola-del-oars-multidimensional-14256	NA	https://cdn.publisher.gn1.link/ggaging.com/pdf/v2n4a02.pdf Characteristics of instruments of functional assessment in the elderly persons living within the community in Brazil Características de instrumentos de avaliação funcional em idosos residentes na comunidade no Brasil	Assessment of the functional status of elderly subjects in Qassim Region, Saudi Arabia Mohammed A. Al-Mahadi, FFCM KFU, and Ahmed G. Elzubier, MRCPView all authors and affiliations Volume 117, Issue 5 https://doi.org/10.1177/146642409711700509	Ferring D, Windle G, Heiss C, et al. (2001). European Study of Adult Well-Being (ESAW): Comparative report on physical health and functional status. EU 5th Framework Project, Contract number: QLK6-CT-2001-00280
5	Can you go shopping for groceries or clothes (assuming you have transportation)…	OARS Multidimensional Functional Assessment	Chiu HC, Chen YC, Mau LW, et al. An evaluation of the reliability and validity of the Chinese-version OARS Multidimensional Functional Assessment Questionnaire. Chinese J Public	https://www.elsevier.es/es-revista-atencion-primaria-27-articulo-version-espanola-del-oars-multidimensional-14256	NA	https://cdn.publisher.gn1.link/ggaging.com/pdf/v2n4a02.pdf Characteristics of instruments of functional assessment in the elderly persons living within the community in Brazil Características de instrumentos de avaliação funcional	Assessment of the functional status of elderly subjects in Qassim Region, Saudi Arabia Mohammed A. Al-Mahadi, FFCM KFU, and Ahmed G. Elzubier, MRCP. View all authors and affiliations Volume 117, Issue 5 https://doi.org/10.1177/146642409711700509	Ferring D, Windle G, Heiss C, et al. (2001). European Study of Adult Well-Being (ESAW): Comparative report on physical health and functional status. EU 5th Framework Project, Contract number: QLK6-CT-2001-00280

#	Description	English language source	Chinese	Spanish	Hindi	Portuguese	Arabic	French
			Health. 1997;16 :119–132.			em idosos residentes na comunidade no Brasil		
6	Can you prepare your own meals…	OARS Multidimensional Functional Assessment	Chiu HC, Chen YC, Mau LW, et al. An evaluation of the reliability and validity of the Chinese-version OARS Multidimensional Functional Assessment Questionnaire. Chinese J Public Health. 1997;16 :119–132.	https://www.elsevier.es/es-revista-atencion-primaria-27-articulo-version-espanola-del-oars-multidimensional-14256	NA	https://cdn.publisher.gn1.link/ggaging.com/pdf/v2n4a02.pdf Characteristics of instruments of functional assessment in the elderly persons living within the community in Brazil Características de instrumentos de avaliação funcional em idosos residentes na comunidade no Brasil	Assessment of the functional status of elderly subjects in Qassim Region, Saudi Arabia Mohammed A. Al-Mahadi, FFCM KFU, and Ahmed G. Elzubier, MRCP. View all authors and affiliations Volume 117, Issue 5<br><br>https://doi.org/10.1177/146642409711700509	Ferring D, Windle G, Heiss C, et al. (2001). European Study of Adult Well-Being (ESAW): Comparative report on physical health and functional status. EU 5th Framework Project, Contract number: QLK6-CT-2001-00280
7	Can you do your housework…	OARS Multidimensional Functional Assessment	Chiu HC, Chen YC, Mau LW, et al. An evaluation of the reliability and validity of the Chinese-version OARS Multidimensional Functional Assessment Questionnaire. Chinese J Public Health. 1997;16 :119–132.	https://www.elsevier.es/es-revista-atencion-primaria-27-articulo-version-espanola-del-oars-multidimensional-14256	NA	https://cdn.publisher.gn1.link/ggaging.com/pdf/v2n4a02.pdf Characteristics of instruments of functional assessment in the elderly persons living within the community in Brazil Características de instrumentos de avaliação funcional em idosos residentes	Assessment of the functional status of elderly subjects in Qassim Region, Saudi Arabia Mohammed A. Al-Mahadi, FFCM KFU, and Ahmed G. Elzubier, MRCP. View all authors and affiliations Volume 117, Issue 5 https://doi.org/10.1177/146642409711700509	Ferring D, Windle G, Heiss C, et al. (2001). European Study of Adult Well-Being (ESAW): Comparative report on physical health and functional status. EU 5th Framework Project, Contract number: QLK6-CT-2001-00280

#	Description	English language source	Chinese	Spanish	Hindi	Portuguese	Arabic	French
						na comunidade no Brasil		
8	Can you take your own medicines…	OARS Multidimensional Functional Assessment	Chiu HC, Chen YC, Mau LW, et al. An evaluation of the reliability and validity of the Chinese-version OARS Multidimensional Functional Assessment Questionnaire. Chinese J Public Health. 1997;16 :119–132.	https://www.elsevier.es/es-revista-atencion-primaria-27-articulo-version-espanola-del-oars-multidimensional-14256	NA	https://cdn.publisher.gn1.link/ggaging.com/pdf/v2n4a02.pdf Characteristics of instruments of functional assessment in the elderly persons living within the community in Brazil Características de instrumentos de avaliação funcional em idosos residentes na comunidade no Brasil	Assessment of the functional status of elderly subjects in Qassim Region, Saudi Arabia Mohammed A. Al-Mahadi, FFCM KFU, and Ahmed G. Elzubier, MRCPView all authors and affiliations Volume 117, Issue 5 https://doi.org/10.1177/146642409711700509	Ferring D, Windle G, Heiss C, et al. (2001). European Study of Adult Well-Being (ESAW): Comparative report on physical health and functional status. EU 5th Framework Project, Contract number: QLK6-CT-2001-00280
9	Can you handle your own money…	OARS Multidimensional Functional Assessment	Chiu HC, Chen YC, Mau LW, et al. An evaluation of the reliability and validity of the Chinese-version OARS Multidimensional Functional Assessment Questionnaire. Chinese J Public Health. 1997;16 :119–132.	https://www.elsevier.es/es-revista-atencion-primaria-27-articulo-version-espanola-del-oars-multidimensional-14256	NA	https://cdn.publisher.gn1.link/ggaging.com/pdf/v2n4a02.pdf Characteristics of instruments of functional assessment in the elderly persons living within the community in Brazil Características de instrumentos de avaliação funcional em idosos residentes	Assessment of the functional status of elderly subjects in Qassim Region, Saudi Arabia Mohammed A. Al-Mahadi, FFCM KFU, and Ahmed G. Elzubier, MRCPView all authors and affiliations Volume 117, Issue 5 https://doi.org/10.1177/146642409711700509	Ferring D, Windle G, Heiss C, et al. (2001). European Study of Adult Well-Being (ESAW): Comparative report on physical health and functional status. EU 5th Framework Project, Contract number: QLK6-CT-2001-00280

#	Description	English language source	Chinese	Spanish	Hindi	Portuguese	Arabic	French
						na comunidade no Brasil		
10	Can you get in and out of bed...	OARS Multidimensional Functional Assessment	Chiu HC, Chen YC, Mau LW, et al. An evaluation of the reliability and validity of the Chinese-version OARS Multidimensional Functional Assessment Questionnaire. Chinese J Public Health. 1997;16 :119–132.	https://www.elsevier.es/es-revista-atencion-primaria-27-articulo-version-espanola-del-oars-multidimensional-14256	NA	https://cdn.publisher.gn1.link/ggaging.com/pdf/v2n4a02.pdf Characteristics of instruments of functional assessment in the elderly persons living within the community in Brazil Características de instrumentos de avaliação funcional em idosos residentes na comunidade no Brasil	Assessment of the functional status of elderly subjects in Qassim Region, Saudi Arabia Mohammed A. Al-Mahadi, FFCM KFU, and Ahmed G. Elzubier, MRCPView all authors and affiliations Volume 117, Issue 5 https://doi.org/10.1177/146642409711700509	Ferring D, Windle G, Heiss C, et al. (2001). European Study of Adult Well-Being (ESAW): Comparative report on physical health and functional status. EU 5th Framework Project, Contract number: QLK6-CT-2001-00280
11	Can you dress and undress yourself...	OARS Multidimensional Functional Assessment	Chiu HC, Chen YC, Mau LW, et al. An evaluation of the reliability and validity of the Chinese-version OARS Multidimensional Functional Assessment Questionnaire. Chinese J Public Health. 1997;16 :119–132.	https://www.elsevier.es/es-revista-atencion-primaria-27-articulo-version-espanola-del-oars-multidimensional-14256	NA	https://cdn.publisher.gn1.link/ggaging.com/pdf/v2n4a02.pdf Characteristics of instruments of functional assessment in the elderly persons living within the community in Brazil Características de instrumentos de avaliação funcional em idosos residentes	Assessment of the functional status of elderly subjects in Qassim Region, Saudi Arabia Mohammed A. Al-Mahadi, FFCM KFU, and Ahmed G. Elzubier, MRCPView all authors and affiliations Volume 117, Issue 5 https://doi.org/10.1177/146642409711700509	Ferring D, Windle G, Heiss C, et al. (2001). European Study of Adult Well-Being (ESAW): Comparative report on physical health and functional status. EU 5th Framework Project, Contract number: QLK6-CT-2001-00280

#	Description	English language source	Chinese	Spanish	Hindi	Portuguese	Arabic	French
						na comunidade no Brasil		
12	Can you take a bath or shower…	OARS Multidimensional Functional Assessment	Chiu HC, Chen YC, Mau LW, et al. An evaluation of the reliability and validity of the Chinese-version OARS Multidimensional Functional Assessment Questionnaire. Chinese J Public Health. 1997;16 :119–132.	https://www.elsevier.es/es-revista-atencion-primaria-27-articulo-version-espanola-del-oars-multidimensional-14256	NA	https://cdn.publisher.gn1.link/ggaging.com/pdf/v2n4a02.pdf Characteristics of instruments of functional assessment in the elderly persons living within the community in Brazil Características de instrumentos de avaliação funcional em idosos residentes na comunidade no Brasil	Assessment of the functional status of elderly subjects in Qassim Region, Saudi Arabia Mohammed A. Al-Mahadi, FFCM KFU, and Ahmed G. Elzubier, MRCPView all authors and affiliations Volume 117, Issue 5 https://doi.org/10.1177/146642409711700509	Ferring D, Windle G, Heiss C, et al. (2001). European Study of Adult Well-Being (ESAW): Comparative report on physical health and functional status. EU 5th Framework Project, Contract number: QLK6-CT-2001-00280
13	During the past 4 weeks, how much of the time has your physical health or emotional problems interfered with your social activities (like visiting friends, relatives, etc.)?	MOS SF-36	Li, Li, Wang, H. M., & Shen, Y. (2003). Chinese SF-36 Health Survey: translation, cultural adaptation, validation, and normalisation. Journal of Epidemiology and Community Health, 57(4), 259–263.	Alonso J, Prieto L, Anto JM. La versión española del SF-36 Health Survey (Cuestionario de Salud SF-36): un instrumento para la medida de los resultados clínicos. Med Clin (Barc).	Indian Journal of Community Medicine 38(1):p 22-26, Jan–Mar 2013. | DOI: 10.4103/0970-0218.106623	Brazilian-Portuguese version of the SF-36. A reliable and valid quality of life outcome measure January 1999Revista Brasileira de Reumatologia 39(3):143-150 January 199939(3):143-150	https://www.rand.org/content/dam/rand/pubs/papers/2009/P7995.pdf	J Clin Epidemiol . 1998 Nov;51(11):1013-23. doi: 10.1016/s0895-4356(98)00093-6.

#	Description	English language source	Chinese	Spanish	Hindi	Portuguese	Arabic	French
				1995;104:771-6.				
14	How is your eyesight (with glasses or contacts)?	Direct question (self-rating of eyesight)	Y	Y	Y	Y	Y	Y
15	How is your hearing (with a hearing aid, if needed)?	Direct question (self-rating of hearing)	Y	Y	Y	Y	Y	Y
16	Are you basically satisfied with your life? (and 4 more questions)	GDS	Clinical Validation of the Geriatric Depression Scale (GDS): Chinese Version Alfred Cheung-Ming Chan, PhDView all authors and affiliations Volume 8, Issue 2 https://doi.org/10.1177/089826439600800205	DOI: 10.1016/S0014-2565(07)73477-X Validación de la versión española de 5 y 15 ítems de la Escala de Depresión Geriátrica en personas mayores en Atención Primaria Validation of 5 and 15 items Spanish version of the geriatric depression scale in elderly subjects in Primary Health Care	Int J Geriatr Psychiatry. 1999 Oct;14(10):807-20.	Arq. Neuro-Psiquiatr. 57 (2B) • Jun 1999 • https://doi.org/10.1590/S0004-282X1999000300013	Int Psychogeriatr. 2008 Jun;20(3):571-81. doi: 10.1017/S1041610208006741. Epub 2008 Feb 21. Validation of the Arabic version of the short Geriatric Depression Scale (GDS-15)	https://www.has-sante.fr/upload/docs/application/pdf/2015-02/argumentaire_art_53_depression_sujets_ages_vf_2015-02-16_15-33-53_53.pdf

#	Description	English language source	Chinese	Spanish	Hindi	Portuguese	Arabic	French
				setting. R. Ortega Orcosa, MA. Salinero Fortb, A. Kazemzadeh Khajouia, S. Vidal Aparicioa, R. de Dios del Vallec				
17	KINDS OF SUPPORT	MOS SSS	Res Nurs Health. 2004 Apr;27(2):135-43. doi: 10.1002/nur.20008.	Ahumada L, Castillo J, Muñoz E, Moruno I. Validación del cuestionario MOS de apoyo social en atención primaria. Med Fam. (2005) 6: 10–8.	Not available	Fachado A, Martinez A, Villalva C, Pereira M. Adaptação cultural e validação da versão Portuguesa questionário medical outcomes study social support survey (MOS-SSS) [cultural adaptation and validation of the medical outcomes study social support survey questionnaire (MOS-SSS)]. Acta Méd Port. (2007) 20:525–34.	Dafaalla M, Farah A, Bashir S, Khalil A, Abdulhamid R, Mokhtar M, et al. Validity and reliability of Arabic MOS social support survey. SpringerPlus. (2016) 5:1306. doi: 10.1186/s40064-016-2960-4	Can J Cardiol . 2005 Aug;21(10):867-73.
18	IN the past 7 days… (anxiety questions)	PROMIS Anxiety	Y Available from PROMIS https://www.healthmeasures.n	Y Available from PROMIS https://www. healthmeasur	Y Available from PROMIS https://www. healthmeasur	Y Available from PROMIS https://www.health measures.net/explor	Y Available from PROMIS https://www.healthmeasures.net/explore-measurement-	Y Available from PROMIS https://www.health measures.net/explo

#	Description	English language source	Chinese	Spanish	Hindi	Portuguese	Arabic	French
			et/explore-measurement-systems/promis/intro-to-promis/available-translations/117-available-translations	es.net/explore-measurement-systems/promis/intro-to-promis/available-translations/117-available-translations	es.net/explore-measurement-systems/promis/intro-to-promis/available-translations/117-available-translations	e-measurement-systems/promis/intro-to-promis/available-translations/117-available-translations	systems/promis/intro-to-promis/available-translations/117-available-translations	re-measurement-systems/promis/intro-to-promis/available-translations/117-available-translations
19	Your Health	Comorbidity List	Y	Y	Y	Y	Y	Y
20	How much weight have you lost in the past 3 months?	MNA	https://www.mna-elderly.com/sites/default/files/2021-10/mna-mini-chinese.pdf	https://www.mna-elderly.com/sites/default/files/2021-10/mna-mini-spanish.pdf	https://www.mna-elderly.com/sites/default/files/2021-10/mna-mini-hindi.pdf	https://www.mna-elderly.com/sites/default/files/2021-10/mna-mini-portuguese-brazil.pdf	https://www.mna-elderly.com/sites/default/files/2021-10/mna-mini-arabic.pdf	https://www.mna-elderly.com/sites/default/files/2021-10/mna-mini-french.pdf
21	Gait Speed	Gait speed measurement, general instructions	Y	Y	Y	Y	Y	Y
22	Cognitive Screening	Mini-Cog©	https://mini-cog.com/wp-content/uploads/2022/09/CHINESE-Standardized-Mini-Cog-1-19-16-ZHO-TW_v1.pdf	https://mini-cog.com/wp-content/uploads/2022/09/SPANISH-Mini-Cog.pdf	https://mini-cog.com/wp-content/uploads/2023/05/mini-cog1-Hindi.pdf	https://mini-cog.com/wp-content/uploads/2022/09/PORTUGUESE-Mini-Cog-in-Portuguese.pdf	https://mini-cog.com/wp-content/uploads/2022/09/ARABIC-Standardized-Mini-Cog-in-Arabic.pdf	https://mini-cog.com/wp-content/uploads/2022/09/FRENCH-Standardized-Mini-Cog-1-19-16-FR_v1-hi-3.pdf

#	Description	English language source	Chinese	Spanish	Hindi	Portuguese	Arabic	French
23	Chemotherapy Toxicity Calculator	CARG Toxicity Tool	Y	Y	N	https://pubmed.ncbi.nlm.nih.gov/31401205/	Y	Y

Abbreviations: CARG, Cancer and Aging Research Group; GDS, Geriatric Depression Scale; EU, European Union; MNA, Mini Nutritional Assessment; MOS SF-36, Medical Outcomes Study Short Form-36; MOS SSS, Medical Outcomes Study Social Support Survey; N, no; NA, not available; OARS, Older Americans Resources and Services, PROMIS, Patient-Reported Outcomes Measurement Information System; QoL, Quality of Life; Y, yes

Table 3. ePrognosis tools with national origin of population

ePrognosis	Population	Available translations				Citation	Countries
**Hospitalized**							
Walter Index	Hospitalized adults age 70 and older	English	Spanish	French	Portuguese	Walter LC, Brand RJ, Counsell SR, Palmer RM, Landefeld CS, Fortinsky RH, Covinsky KE. Development and validation of a prognostic index for 1-year mortality in older adults after hospitalization. JAMA 2001;285:2987-2994.	US
Teno 1 Year Help Model Index	Medicine and ICU patients aged 80 years and older	English				Teno JM, Harrell Jr FE, Knaus W, Phillips RS, Wu AW, Connors Jr A, Wenger NS, Wagner D, Galanos A, Desbiens NA, Lynn J. Prediction of survival for older hospitalized patients: The HELP survival model. JAGS 2000;48(5 Suppl):S16-24	US
Pilotto Index	Hospitalized elders aged 65 and older	English				San Carlo D, D'Onofrio G, Franceschi M, Scarcelli c, Niro V, Addante F, Copetti M, Ferrucci L, Fontanna L, Pilotto A. Validation of a modified-multidimensional prognostic index (m-MPI) including the mini nutritional assesment short-term (MNA-SF) for the prediction of one-year mortality in hospitalized elderly patients. J Nurr Health Aging. Epu 7 Octorber 2010.	US
Palliative Performance Scale	Hospitalized patients with palliative care consultation	English				Anderson F, Downing GM, Hill J, Casorso L, Lerch N. Palliative Performance scale (PPS): a new tool. J Pall Care, 12(1): 5-11	Canada
Levine Index	Older patients discharged from general medicine service at a teaching hospital	English				Levine SK, Sachs GA, Jin L, Meltzer D. A prognostic model for 1-year mortality in older adults after hospital discharge. Am J Med. 2007 May;120(5):455-60.	US
Inouye 1 year index	Hospitalized individuals aged 70 years and older	English				Inouye SK, Bogardus ST, Vitagliano G, Inouye MM, Williams CS, Grady JN, Scinto JD. Burden of illness score for elderly persons: Risk	US

						adjustment incorporating the cumulative impact of diseases, physiologic abnormalities, and functional impairments. Medical Care. 2003;41(1):70-83.	
Dramé 2 year Index	Hospitalized adults aged 75 years and older	English				Dramé M, Novella JL, Lang PO, Somme D, Jovenin N, Lanièce I, Couturier P, Heitz D, Gauvain JB, Voisin T, De Wazières B, Gonthier R, Ankri J, Heandel C, Saint-Jean O, Blanchard F, Jolly D. Derivation and validation of a mortality-risk index from a cohort of frail elderly patients hospitalized in medical wards via emergencies: The SAFES study. Eur J Epidemiol. 2008;23:780-791.	France
Di Bari 1 year Silver Code	Adults aged 75 and older admitted to the emergency department	English				Di Bari M, Balzi D, Roberts AT, Barchielli A, Fumagalli S, Ungar A, Bandinelli S, De Alfieri W, Gabbani L, Marchionni N. Prognostic stratification of older persons based on simple administrative data: Development and validation of the "silver code," to be used in emergency department triage. J Gerontol A Biol Sci Med Sci. 2010 Feb;65(2):159-164.	Italy
**Living in a Nursing Home**							
Porock Index	Nursing home residents	English				Porock D, Parker-Oliver D, Petroski GF, Rantz M. The MDS mortality risk index: The evolution of a method for predicting 6-month mortality in nursing home residents. BMC Research Notes, 2010 July 16:3:200-208.	US, England
Mitchell Index	Nursing home adults aged 65 and older	English				Mitchell SL, Miller SC, Teno JM, Kiely DK, Davis RB, Shaffer ML. Prediction of 6-Month Survival of Nursing Home Residents With Advanced Dementia Using ADEPT vs Hospice Eligibility Guidelines. JAMA. 2010;304(17):1929-1935. doi:10.1001/jama.2010.1572.	US

Flacker 1 Year Newly Admitted Revised Index	Newly admitted nursing home residents aged 65 and old	English				Flacker JM, Kiely DK. Mortality-related factors and 1-year survival in nursing home residents. JAGS. 2003;51:213-221.	US
Flacker 1 Year Long Stay Revised Index	Long stay (1 year or longer) nursing home residents aged 65 and older	English				Flacker JM, Kiely DK. Mortality-related factors and 1-year survival in nursing home residents. JAGS. 2003;51:213-221.	US
**Living in the Community**							
Multimorbidity-Weighted Index	Community-dwelling adults aged 54-89 years.	English				Wei MY, Kawachi I, Okereke OI, Mukamal KJ. Diverse Cumulative Impact of Chronic Diseases on Physical Health-Related Quality of Life: Implications for a Measure of Multimorbidity. Am J Epidemiol. 2016 Sep 1;184(5):357-65.	US
Suemoto Index	Community-dwelling adults aged 60 and older	English	Spanish	French	Portuguese	Suemoto CK, Ueda P, Beltrán-Sánchez, Lebrão ML, Duarte YA, Wong R, Danaei G. Development and Validation of a 10-Year Mortality Prediction Model: Meta-Analysis of Individual Participant Data From Five Cohorts of Older Adults in Developed and Developing Countries. J Gerontol A Biol Sci Med Sci. 2016 Aug 13. pii: glw166. [Epub ahead of print]	US, Brazil, Mexico, England
Schonberg Index	Community-dwelling adults aged 65 and older	English				Schonberg MA, Davis RB, McCarthy EP, and Marcantonio ER. Index to predict 5-year mortality of community dwelling adults aged 65 an older using data from the National Health Interview Survey. J Gen Intern Med. 2009;24(10):1115-1022.	US
Palliative Performance Scale	Outpatients, 81% with metastatic cancer. Please use with caution in non-cancer patients. For non-cancer patients, the Walter Index may be more appropriate.	English				Anderson F, Downing GM, Hill J, Casorso L, Lerch N. Palliative Performance scale (PPS): a new tool. J Pall Care, 12(1): 5-11	Canada

Palliative Performance Scale	Community based hospice	English				Anderson F, Downing GM, Hill J, Casorso L, Lerch N. Palliative Performance scale (PPS): a new tool. J Pall Care, 12(1): 5-11	Canada
Mazzaglia Index	Community dwelling patients aged 65 years and older	English				Mazzaglia GM, Roti L, Corsini G, Colombini A, Maciocco G, Marchionni N, Buiatti E, Ferrucci L, Di Bari M. Screening of older community-dwelling people at risk for death and hospitalization: The Assistenza Socio-Sanitaria in Italia Project. JAGS. 2007;55:1955-1960.	Italy
Lee Schonberg Index	Community dwelling adults aged 50 and older	English	Spanish	French	Portuguese	Lee SJ, Lindquist K, Segal MR, Covinsky KE. Development and validation of a prognostic index for 4-year mortality in older adults. JAMA. 2006 Feb 15;295(7):801-808.	US
Comprehensive Prognostic Tool for Adults ≥ 70		English				Lee AK, Diaz-Ramirez LG, Boscardin WJ, Smith AK, Lee SJ. A Comprehensive Prognostic Tool for Older Adults: Predicting Death, ADL Disability, and Walking Disability Simultaneously. Journal of the American Geriatrics Society. DOI: 10.1111/jgs.17932	US
Gagne Index	Community-dwelling adults aged 65 years and older	English				Gagne JJ, Glynn RJ, Avorn J, Levin R, Schneeweiss S. A combined comorbidity score predicted mortality in elderly patients better than existing scores. J Clin Epidemiol. Jul 2011;64(7):749-759.	US
Models to predict need for nursing home level of care in community-dwelling older adults with dementia	Community dwelling older adults aged 65 years and older with dementia	English				Deardorff WJ, Jeon SY, Barnes DE, Boscardin WJ, Langa KM, Covinsky KE, Mitchell SL, Lee SJ, Smith AK. Development and External Validation of Models to Predict Need for Nursing Home Level of Care in Community-Dwelling Older Adults With Dementia. JAMA Intern Med. 2024 Jan 1;184(1):81-91. doi: 10.1001/jamainternmed.2023.6548.	US

Mortality Risk Calculator for Community-Dwelling Older Adults with Dementia	Community-dwelling older adults aged 65 years and older with dementia	English				Deardorff WJ, Barnes DE, Jeon SY, Boscardin WJ, Langa KM, Covinsky KE, Mitchell SL, Whitlock EL, Smith AK, Lee SJ. Development and external validation of a mortality prediction model for community-dwelling older adults with dementia. JAMA Intern Med. 2022;182(11):1161-1170. PMID 36156062.	US
Carey 3 Year Index	Community dwelling nursing home eligible adults aged 55 years and older	English				Carey EC, Covinsky KE, Li-Yung L, Eng C, Sands LP, Walter LC. Prediction of mortality in community-living frail elderly people with long-term care needs. JAGS. 2008;56(1):68-75.	US
Carey 2 Year Index	Community dwelling adults 70 years and older	English				Carey CE, Walter LC, Lindquist K, Covinsky KE. Development and validation of a functional morbidity index to predict mortality in community-dwelling elders. J Gen Intern Med. 2004:19:1027-33.	US
Breast Cancer Risk Assessment for Postmenopausal Women Age 55 or Older with Consideration of Competing Mortality Risks	Postmenopausal women age 55 years and older (with no history of breast cancer or genetic mutations that increase their risk of breast cancer)	English				Schonberg MA, Wolfson EA, Eliassen AH, Bertrand KA, Shvetsov YB, Rosner BA, Palmer JR, Ngo LH. A model for predicting both breast cancer risk and non-breast cancer death among women > 55 years old. Breast Cancer Res. 2023 Jan 24;25(1):8. doi: 10.1186/s13058-023-01605-8.	US

Abbreviations: ICU, intensive care unit; US, United States

*Source: ePrognosis

Data Supplement 2: Search Strategy String and Dates

Search strategy

Note: Searches 1-4 of PubMed. Searches 1-3 were all combined with terms for global health and resource-constrained settings (listed below in lieu of repeating with each search). Search 5 of Google Scholar.

Search 1: PubMed search for randomized studies from 2022 January– 2024 June 14 in resource-constrained settings, humans limit. Geriatric Assessment, cancer. June 2024.

(("geriatric"[All Fields] AND "assessment"[tw] AND "cancer"[All Fields] AND "random*" [tw]) AND

Search 2: PubMed Search for any study design in resource-constrained settings, Geriatric Assessment, cancer. 2022 January – 2024 June 24.

Search String (("geriatric"[All Fields] AND "assessment"[tw] AND "cancer"[All Fields]) AND

Search 3: PubMed Search from 2022 January – 2024 June 24 for implementation in resource-constrained settings, with Humans limit). Geriatric Assessment and Implementation, with or without cancer. June 2024.

Search String: ((Geriatric Assessment [MesH] AND Implementation[tw]) AND˜

Search 4: PubMed search for Systematic Reviews since 2016 (2022 January – 2024 June 28) regarding prognostic or predictive overall mortality cancer. Human, Systematic Review. Filters: Human and SRs

Search string (("Mortality"[Majr]) AND "Neoplasms"[MeSH]) AND ("Prognosis"[MeSH] OR "Predictive Value of Tests"[MAJR]) AND (2016[PDAT]:3000[PDAT])

Search 5: Google Scholar search for Systematic Reviews from 2022-2024 on prognostic or predictive studies, overall cancer mortality, humans limit.

Search String ("Systematic Review" AND "Cancer Mortality" AND (2022:3000) AND Human)

AND ("Developing Countries"[Mesh] OR "global health"[MH] OR "Vulnerable Populations"[MH] OR "poverty"[MeSH Terms] OR "Medically Underserved Area"[MH] OR "Health Disparate Minority and Vulnerable Populations"[Mesh] OR "developing country"[TIAB] OR "low and middle income countries"[TIAB] OR LMIC[TIAB] OR "south america"[MH] OR "latin America"[MH] OR "Asia, Central"[MH] OR "Asia, Southeastern"[MH] OR "Asia, Western"[MH] OR "afghanistan"[MeSH Terms] OR "albania"[MeSH Terms] OR "algeria"[MeSH Terms] OR "angola"[MeSH Terms] OR "Antigua and Barbuda"[MH] OR Barbuda[TIAB] OR "argentina"[MeSH Terms] OR "armenia"[MeSH Terms] OR Armenian[TIAB] OR Aruba[TIAB] OR "azerbaijan"[MeSH Terms] OR "bahrain"[MeSH Terms] OR "bangladesh"[MeSH Terms] OR "barbados"[MeSH Terms] OR "benin"[MeSH Terms] OR "belize"[MeSH Terms] OR "bhutan"[MeSH Terms] OR "bolivia"[MeSH Terms] OR "botswana"[MeSH Terms] OR

"brazil"[MeSH Terms] OR "Burkina Faso"[MH] OR "Burkina Fasso"[TIAB] OR "burundi"[MeSH Terms] OR Urundi[TIAB] OR "cambodia"[MeSH Terms] OR "Khmer Republic"[TIAB] OR Kampuchea[TIAB] OR "cameroon"[MeSH Terms] OR Cameroons[TIAB] OR Cameron[TIAB] OR "Cape Verde"[MH] OR "Central African Republic"[MH] OR "chad"[MeSH Terms] OR "chile"[MeSH Terms] OR "china"[MeSH Terms] OR "colombia"[MeSH Terms] OR "comoros"[MeSH Terms] OR "Comoro Islands"[TIAB] OR Comores[TIAB] OR Mayotte[TIAB] OR "congo"[MeSH Terms] OR Zaire[TIAB] OR "Costa Rica"[MH] OR "Cote d'Ivoire"[MH] OR "Ivory Coast"[TIAB] OR "djibouti"[MeSH Terms] OR "French Somaliland"[TIAB] OR "dominica"[MeSH Terms] OR "Dominican Republic"[MH] OR "East Timor"[TIAB] OR "Timor Leste"[MH] OR "ecuador"[MeSH Terms] OR "egypt"[MeSH Terms] OR "United Arab Republic"[TIAB] OR "El Salvador"[MH] OR "eritrea"[MeSH Terms] OR "ethiopia"[MeSH Terms] OR "fiji"[MeSH Terms] OR "gabon"[MeSH Terms] OR "Gabonese Republic"[TIAB] OR "gambia"[MeSH Terms] OR Gaza[TIAB] OR ("georgia"[MeSH Terms] OR "georgia (republic)"[MeSH Terms]) OR "ghana"[MeSH Terms] OR "grenada"[MeSH Terms] OR "guatemala"[MeSH Terms] OR "guinea"[MeSH Terms] OR "Guinea-Bissau"[MH] OR Guiana[TIAB] OR "guyana"[MeSH Terms] OR "haiti"[MeSH Terms] OR "honduras"[MeSH Terms] OR "india"[MeSH Terms] OR "Indian Ocean Islands"[MH] OR Maldives[TIAB] OR "indonesia"[MeSH Terms] OR "kenya"[MeSH Terms] OR Kiribati[TIAB] OR "Lao PDR"[TIAB] OR "laos"[MeSH Terms] OR "lesotho"[MeSH Terms] OR Basutoland[TIAB] OR "liberia"[MeSH Terms] OR "libya"[MeSH Terms] OR "madagascar"[MeSH Terms] OR "Malagasy Republic"[TIAB] OR Sabah[TIAB] OR Sarawak[TIAB] OR "malawi"[MeSH Terms] OR Nyasaland[TIAB] OR "mali"[MeSH Terms] OR "malta"[MeSH Terms] OR "Marshall Islands"[TIAB] OR "mauritania"[MeSH Terms] OR "mauritius"[MeSH Terms] OR "Agalega Islands"[TIAB] OR "mexico"[MeSH Terms] OR "micronesia"[MeSH Terms] OR "moldova"[MeSH Terms] OR Moldovia[TIAB] OR Moldovian[TIAB] OR "mongolia"[MeSH Terms] OR "montenegro"[MeSH Terms] OR "morocco"[MeSH Terms] OR Ifni[TIAB] OR "mozambique"[MeSH Terms] OR "myanmar"[MeSH Terms] OR Myanma[TIAB] OR Burma[TIAB] OR "namibia"[MeSH Terms] OR "nepal"[MeSH Terms] OR "Netherlands Antilles"[MH] OR "New Caledonia"[MH] OR "nicaragua"[MeSH Terms] OR "niger"[MeSH Terms] OR "nigeria"[MeSH Terms] OR "Northern Mariana Islands"[TIAB] OR "oman"[MeSH Terms] OR Muscat[TIAB] OR "pakistan"[MeSH Terms] OR "palau"[MeSH Terms] OR Palestine[TIAB] OR "panama"[MeSH Terms] OR "paraguay"[MeSH Terms] OR "peru"[MeSH Terms] OR "philippines"[MeSH Terms] OR Philipines[TIAB] OR Phillipines[TIAB] OR Phillippines[TIAB] OR "rwanda"[MeSH Terms] OR Ruanda[TIAB] OR "Saint Kitts and Nevis"[MH] OR "St Kitts"[TIAB] OR Nevis[TIAB] OR "Saint Lucia"[MH] OR "St Lucia"[TIAB] OR "Saint Vincent and the Grenadines"[MH] OR "St Vincent"[TIAB] OR Grenadines[TIAB] OR "samoa"[MeSH Terms] OR "Samoan Islands"[TIAB] OR "Sao Tome"[TIAB] OR "Saudi Arabia"[MH] OR "senegal"[MeSH Terms] OR "serbia"[MeSH Terms] OR "Sierra Leone"[MH] OR "slovenia"[MeSH Terms] OR "Sri Lanka"[MH] OR Ceylon[TIAB] OR "Solomon Islands"[TIAB] OR "somalia"[MeSH Terms] OR "sudan"[MeSH Terms] OR "suriname"[MeSH Terms] OR Surinam[TIAB] OR "swaziland"[MeSH Terms] OR "tajikistan"[MeSH Terms] OR Tadzhikistan[TIAB] OR Tadjikistan[TIAB] OR Tadzhik[TIAB] OR "tanzania"[MeSH Terms] OR "thailand"[MeSH Terms] OR "togo"[MeSH Terms] OR "Togolese Republic"[TIAB] OR "tonga"[MeSH Terms] OR "Trinidad and Tobago"[MH] OR "tunisia"[MeSH Terms] OR "turkey"[MeSH Terms] OR "turkmenistan"[MeSH Terms] OR "kazakhstan"[MeSH Terms] OR Turkmen[TIAB] OR "kyrgyzstan"[MeSH Terms] OR "uzbekistan"[MeSH Terms] OR "uganda"[MeSH Terms] OR "ukraine"[MeSH Terms] OR "uruguay"[MeSH Terms] OR "vanuatu"[MeSH Terms] OR "New Hebrides"[TIAB] OR "venezuela"[MeSH Terms] OR "vietnam"[MeSH Terms] OR "Viet Nam"[TIAB] OR

"zambia"[MeSH Terms] OR "zimbabwe"[MeSH Terms] OR "Africa, Northern"[All Fields] OR "Northern Africa"[TIAB] OR "North Africa"[TIAB] OR "Africa South of the Sahara"[MH] OR "sub-Saharan Africa"[TIAB] OR "subsaharan Africa"[TIAB] OR "Africa, Central"[MH] OR "central Africa"[TIAB] OR "Africa, Eastern"[MH] OR "east Africa"[TIAB] OR "Africa, Southern"[MH] OR "southern Africa"[TIAB] OR "Africa, Western"[MH] OR "western Africa"[TIAB] OR "west africa"[TIAB] OR "Caribbean Region"[MH] OR "West Indies"[MH] OR Caribbean[TIAB] OR "Central America"[MH] OR "Panama Canal Zone"[MH] OR "French Guiana"[MH] OR "borneo"[MeSH Terms] OR "brunei"[MeSH Terms] OR "Mekong Valley"[MH] OR "mekong delta"[TIAB] OR "Republic of Congo"[TIAB] OR "Congo-Brazzaville"[TIAB] OR "Democratic Republic of the Congo"[MH] OR DRC[TIAB] OR "Equatorial Guinea"[MH] OR "South Sudan"[MH] OR "South Africa"[MH] OR "cuba"[MeSH Terms]))

Data Supplement 3: Search Yields

Searches 1-5

Search 1. PubMed search for randomized studies from 2022-2024 in resource-constrained settings, humans limit. Geriatric Assessment, cancer. June 2024.

Yield: n=12, 0 relevant

Search 2. PubMed Search for any study design in resource-constrained settings, Geriatric Assessment, cancer. June 2024.

Yield: n=179

Search 3. Implementation, June 2024. PubMed Search from 2022-2024 for implementation in resource-constrained settings, with Humans limit). Geriatric Assessment and Implementation, with or without cancer.

Yield: n=30, 7 potentially relevant, 3 included for review

Search 4. PubMed search for Systematic Reviews since 2016 regarding prognostic or predictive overall mortality cancer. Human, Systematic Review. Filters: Human and SRs.

Yield: n=24, 4 potentially relevant, 0 cited

Search 5. Google Scholar search for Systematic Reviews from 2022-2024 on prognostic or predictive studies, overall cancer mortality, humans limit.

Note: reviewed first 4 pages of results

Yield: n=977, 8 potentially relevant, zero cited

Data Supplement 4: Clinical Questions

(1) What is the role of geriatric assessment (GA) in older adults with cancer to inform specific interventions to improve clinical outcomes in resource-constrained settings?

(2) For older patients who are considering undergoing antineoplastic therapy and other systemic treatments, which GA tools and component elements should clinicians use to predict adverse outcomes (including antineoplastic therapy toxicity and mortality) and guide management in resource-constrained settings?

(3) What general (ie, noncancer-specific) life expectancy data for community-dwelling patients should clinicians consider to estimate mortality and best inform treatment decision making for older patients with cancer in resource-constrained settings?

(4) How should GA be used to guide management of older patients with cancer in resource-constrained settings?

Data Supplement 5: Development of Recommendations Using Formal Consensus

ASCO adapted the modified Delphi approach for formal expert consensus from the Cancer Care Ontario consensus guideline experience in addition to using the ADAPTE process.[10,11] The formal consensus process involved members of the Expert Panel which drafted the recommendations using adaptation of existing guidelines and clinical expertise. The data were considered insufficient to inform evidence-based recommendations for most resource-constrained settings; but were used to help members of the Expert Panel form opinions. The Expert Panel met multiple times to review the recommendations. ASCO then assembled a Consensus Ratings Panel for independent and anonymous voting on draft recommendations, with subsequent revisions by the Co-Chairs of the Expert Panel. The goal was to reach consensus, defined by ASCO as $\geq$75% of raters indicating agreement with a given recommendation. This threshold was met after one round of rating. This methodology is described in further detail elsewhere.[10]

Consensus Round One
The draft recommendations were distributed to the Consensus Ratings Panel to rate their approval with the guideline and recommendations. Comments were invited. A total of 26 members (11 of whom were on the Expert Panel) participated. Greater than or equal to 80% of the responses received a rating of either “agree” or “strongly agree” for each of the 16 questions. The Panel made small changes in response to the comments (5 total questions had 100% rating).

Data Supplement 6: Consensus Panel Member Disclosures

Required for all consensus guidelines

Consensus Panel Members disclosed generally. Their disclosure is not limited to subject matter under consideration in this article.

All relationships are considered compensated. Relationships are self-held unless otherwise noted. I = Immediate Family Member, Inst = My Institution

Name	Last Update	Employment	Leadership	Stock and Other Ownership Interests	Honoraria	Consulting or Advisory Role	Speakers' Bureau	Research Funding	Patents, Royalties, Other Intellectual Property	Expert Testimony	Travel, Accommodations, Expenses	Other Relationship	(OPTIONAL) Uncompensated Relationships
Thierry Alcindor	1/20/2025 11:53:31 AM										SpringWorks Therapeutics		
Daniel A. Anaya	11/18/2024 3:47:55 PM					Fujifilm						Medtronic	
Alexandra Mendes Barreto Arantes	11/12/2024 1:27:21 PM										Merck Roche		
Mehmet Artac	11/12/2024 2:37:39 PM				Amgen<br>Bayer<br>Merck<br>Phizer<br>Roche	Roche							
Pauline Anne P. Cauton	11/18/2024 7:57:30 PM												
Onyema Chido-Amajuoyi	11/26/2024 11:49:40 AM							Pfizer			Abbvie<br>Janssen<br>Roche		
Raul Cordoba	11/12/2024 1:26:36 PM					Abbvie<br>Astra Zeneca<br>BeiGene<br>Celgene/Bristol-Myers Squibb<br>Genmab<br>Incyte<br>Janssen<br>Kite/Gilead<br>Kyowa-Kirin<br>Lilly<br>Regeneron<br>Takeda	Abbvie<br>AstraZeneca<br>Roche	AstraZeneca (Inst)<br>MSD Oncology<br>Novartis (Inst)<br>Roche (Inst)					
Henry L. Gomez	3/3/2025 4:47:48 PM					AstraZeneca	AstraZeneca<br>Bristol-Myers Squibb<br>MSD Oncology<br>Novartis<br>Roche<br>Tecnofarma				Pfizer		

Adeline C. Gonzales	11/13/2024 7:14:35 PM				Astra Zeneca<br>Hi-Esai Pharmaceutical Inc.			Amgen (Inst)<br>AstraZeneca (Inst)<br>Janssen Oncology (Inst)<br>Merck (Inst)<br>Pfizer (Inst)<br>Roche (Inst)						
Gustavo Gössling	11/13/2024 11:18:01 AM													
Coumba Gueye	11/26/2024 10:36:07 AM													

Name	Last Update	Employment	Leadership	Stock and Other Ownership Interests	Honoraria	Consulting or Advisory Role	Speakers' Bureau	Research Funding	Patents, Royalties, Other Intellectual Property	Expert Testimony	Travel, Accommodations, Expenses	Other Relationship	(OPTIONAL) Uncompensated Relationships	(OPTIONAL) Open Payments Link
Muhammad R. Islam	11/12/2024 12:58:30 PM													
Adedayo Joseph	2/12/2025 12:59:12 PM	Lagos University Teaching Hospital				Astra - Zeneca								
Diah Martina	3/3/2025 4:09:37 PM													
Paola C. Montenegro	11/18/2024 12:46:33 PM	Merck Serono (I)			Merck (I)	Tecnofarma	AstraZeneca<br>Bayer<br>Bristol-Myers Squibb<br>Roche			Tecnofarma	Tecnofarma			
Aylen V. Ospina Serrano	11/19/2024 4:50:16 AM										Bristol Myers Squibb<br>Novartis<br>Takeda			
Purvish M. Parikh	11/13/2024 1:05:19 PM													
Sophie Pilleron	11/21/2024 5:01:59 AM											NCCN	International Society of Geriatric Oncology	
Arun Shahi	11/12/2024 11:13:41 PM													
Nisha M. Shariff	11/12/2024 11:34:44 PM					BeiGene	Amgen<br>DKSH<br>Zuellig Pharma				Arcus Biosciences			
Maria del Rosario Sifon	11/15/2024 8:40:18 AM													
Gilliosa Spurrier-Bernard	11/26/2024 9:04:17 AM				Bristol-Myers Squibb<br>MSD									

Kaori Tane	11/20/2024 8:28:11 AM				Chugai Pharma<br>Daiichi Sankyo<br>Eisai<br>lily<br>Pfizer	Chugai Pharma<br>Daiichi Sankyo								
Piotr J. Wysocki	11/12/2024 3:51:03 PM					Astellas Pharma<br>Bristol-Myers Squibb/Medarex<br>Janssen<br>MSD Oncology<br>Pierre Fabre<br>Roche					Gilead Sciences<br>Immunicom<br>Pierre Fabre			

Data Supplement 7: GLIDES Action Verb Glossary

Prescribe	***Prepare***	***Test***	***Monitor***	***Conclude***	***Perform***	***Educate/ Counsel***
add	address	assess	arrange	assess	confine	adhere
adjust	adhere	begin	ascertain	base	ensure	advise
administer	adjust	carry out	assess	conclude	follow	benefit
advance	adopt	check	check	consider	give	clarify
apply	analyze	conduct	conduct	contact	implement	counsel
attempt	attempt	continue	continue	coordinate	include	deliver
avoid	be (aware)	determine	determine	determine	incorporate	discuss
change	become	do	evaluate	diagnose	indicate	educate
choose	begin	evaluate	examine	distinguish	inspect	enable
continue	collect	have	follow up	exclude	offer	encourage
desensitize	continue	identify	have	give (attention)	operate	explain
dilute	dedicate	indicate	include	recognize	perform	have
discontinue	define	measure	institute	recommend	place	help
exercise	develop	need	maintain	respect	receive	identify
improve	encourage	obtain	manage	review	recommend	include
increase	engage	offer	monitor	suspect	relate	incorporate
indicate	ensure	perform	obtain	take (into account)	resect	inform
individualize	establish	prefer	occur	use	reserve	instruct
influence	form	receive	offer	weigh	select	involve
initiate	have	recommend	perform		start	modify
institute	identify	repeat	provide		treat	negotiate
manage	include	require	reassess		undergo	offer
offer	incorporate	reserve	receive		use	promote
order	initiate	restore	recommend			protect
prefer	institute	screen	repeat			provide
prescribe	know	take	require			receive
provide	lead	test	review			recommend
receive	perform	trigger	screen			reinforce
recommend	plan	undergo	warrant			review
reduce	prepare	use				start
repeat	recommend	utilize				support
replace	review					teach
reserve	share					tell
restart	train					use
review	understand					
start	undertake					
suggest	use					
supplement						
taper						
titrate						
treat						
use						
utilize						
warrant						

Dispose	***Document***	***Advocate***	***Examine***	***Inquire***	***Prevent***	***Refer/Consult***
admit	complete	advocate	assess	ask	administer	assess
dispose	document	encourage	auscultate	assess	avoid	conduct
hospitalize	identify	endorse	examine	complete	cleanse	consult
guide	notate	ensure	include	conduct	combine	manage
observe		focus	inspect	gather	continue	obtain
refer		recommend	palpate	include	discard	offer
		work (to)	percuss	incorporate	encourage	recommend
			perform	inquire	give	refer
			use	obtain	immunize	seek
				review	minimize	work (together)
				screen	practice	
				verify	prevent	
					provide	
					receive	
					recommend	
					use	

Glossary from the GuideLines Into Decision Support (GLIDES) project, https://medicine.yale.edu/cmi/glides/.

General Note. ASCO Guideline recommendations (strong or weak) and terminology represent reasonable options for patients depending on clinical circumstances and in the context of individual patient preferences. Recommended care should be accessible to patients whenever possible.

Data Supplement 8:

What is a Geriatric Assessment?

A geriatric assessment is a tool that doctors use to evaluate a person's health and well-being. It looks at your physical function, nutrition, other medical conditions, mental health, thinking and attention ability, current medications, and how much social support you have at home.

In cancer care, a geriatric assessment may be used to understand your current health status and discuss treatment options. As we age, our bodies change. People who are 65 years and older may have specific needs that their health care team wants to understand. This tool will help them to recommend the best treatment plan and provide the right amount of support.

Why are geriatric assessments important?

A geriatric assessment is an important tool because it can uncover information that may not be found during **traditional exams** done during cancer care. This is because people who are 65 years and older often have **other illnesses besides cancer** that influence how treatment side effects could affect them. This information can inform ways the health care team can better support older patients during their treatment and how the **treatment** may need to be adapted to their needs.

Research has shown that geriatric assessments can help doctors understand a person's "functional age" compared to their "chronological age." Chronological age is a person's calendar age. It tells a doctor how much time has passed since a person was born. Functional age describes a person's level of physical functioning in their daily lives. This is useful because not everyone who is the same chronological age has the same physical health or abilities.

As people get older, there can be more differences in health when compared to people of the same chronological age. For example, two people who are in their 70s might have a very different health status from each other. One person might live in their own home, be able to drive, and have a network of family and friends nearby. Another person might live with relatives or in an assisted living facility and have long-distance caregivers; in either setting, both needing help with daily care.

These differences in lifestyle and health have a significant impact on the approach to cancer care and how the health care team supports patients during their cancer experience. That is why geriatric assessments can be so helpful. The assessment is an effort to identify these health differences, use them to make better treatment decisions, and improve your life during and after cancer.

Both the **American Society of Clinical Oncology (ASCO)** and the **International Society of Geriatric Oncology** recommend geriatric assessments for people 65 or older with cancer, particularly people who may need chemotherapy, targeted therapy, or immunotherapy. (Please note these links take you to other websites.)

What does a geriatric assessment include?

A geriatric assessment is not a single test, like a blood pressure reading. Instead, a geriatric assessment is a series of evaluations that can include questionnaires and/or physical evaluations. **ASCO recommends** using the Practical Geriatric Assessment for this purpose, but your doctor may choose to use a different geriatric assessment tool.

A geriatric assessment will typically evaluate:

- Daily living activities
- **Risk of falls**
- **Other medical conditions besides cancer**
- Other medications taken regularly
- Mood, anxiety, and **depression**
- Social activity and support
- Cognition and memory function
- **Nutrition**

To evaluate your daily activities, you may be asked fill out a survey that asks about your ability to prepare your own food and take medications. This is usually done before you see the doctor, such as in the waiting room. To measure your risk for falls, your oncologist may ask a simple question like, "How many falls have you had in the past 6 months or since our last visit?" And details about any other medical conditions may be included as part of your health records. Your oncologist may use these tools together to help inform treatment planning.

Other tools that are specific to older adults undergoing anti-cancer treatment include the G-8 screening tool and some doctors can use locally developed tools, if they include the essential domains, . These tools help predict your chances of experiencing major side effects, also called toxicity, with chemotherapy and can be useful in planning your treatment.

Some of these talks with the doctor may be by telemedicine.

Will I get a score on my geriatric assessment?

A geriatric assessment does not typically include a number score or grade. More likely, your oncologist will discuss your results as part of a larger discussion about **treatment planning**.

For example, patients age 65 or older with cancer have a higher risk of falls. Falls can lead to life-changing injuries that can make it harder to live alone. If you have recently had several falls, your oncologist may recommend **physical therapy** or suggest other options.

How will a geriatric assessment help me?

Cancer treatment always involves certain risks, including possible severe side effects. A geriatric assessment helps your cancer care team understand your current health status and the kind of support you have at home and or in your community. With this information, you and your oncologist can talk about the risks and benefits of a proposed treatment plan and how they fit with your treatment goals and your values. It can also help your care team develop ways to support you better.

Research shows that geriatric assessments have a variety of benefits for older adults with cancer. These include improved communication, fewer unplanned hospital and emergency room visits, and improved quality of life and well-being.

How common are geriatric assessments?

While it is becoming a more common part of cancer care for adults over age 65, not all patients are asked to complete a geriatric assessment. When you are **choosing a doctor for your cancer care**, you might consider asking if they use geriatric assessments in their practice.

Whether or not geriatric assessments are a part of your regular cancer care, be sure to tell your oncologist about your complete medical history, including any recent changes. This includes telling them about:

- Any changes to your health
- New or worsening symptoms of anxiety or depression
- Medications you are taking
- Falls
- Any changes to your living situation or support system

Given that several of the recommendations talk about remote support, the design of a personalized virtual assistant with the following functionalities could be considered:

1) Answers to frequently asked questions (Caregivers and Patients) – for example, see below

2) Presentation of scenarios and cases that require immediate action with their proposals for solutions (action)

3) Explicit, written instructions for appointments, medications, and treatments

4) Medication reminder (to ensure and encourage adherence to treatment)

5) Inclusion of an emergency button aimed at the patient's support persons (responsible family member if they are not the caregiver) or health personnel

This assistant, depending on the deficit, can be aimed at the patient or caregivers.

Questions to ask the health care team

Consider asking your health care team the following:

- Why is it important to provide details about my health history after my cancer diagnosis?
- What tests will I need before my cancer treatment planning is complete?
- What is the goal of each treatment you are recommending? Is it to eliminate the cancer, help me feel better, or both?
- What are the expected side effects of each treatment? How can they be managed or relieved?
- How can I keep myself as healthy and independent as possible during cancer treatment?
- Why is it important to prevent falls at home? How can I minimize my risk of falling?
- Are there cancer rehabilitation services or other care services that can help me?

More Information

NCI's Integrating Geriatric Assessment into Cancer Care: A Conversation with Dr. Supriya Mohile

Note: Modification of content approved by Cancer.Net Editorial Board, 07/2023

References

0. Placeholder of primary publication: Bergerot et al., "Geriatric Assessment: ASCO Global Guideline"

1. Li D, Sun CL, Kim H, et al: Geriatric Assessment-Driven Intervention (GAIN) on Chemotherapy-Related Toxic Effects in Older Adults With Cancer: A Randomized Clinical Trial. JAMA Oncol 7:e214158, 2021
2. Mohile SG, Mohamed MR, Xu H, et al: Evaluation of geriatric assessment and management on the toxic effects of cancer treatment (GAP70+): a cluster-randomised study. Lancet 398:1894-1904, 2021
3. Lund CM, Vistisen KK, Olsen AP, et al: The effect of geriatric intervention in frail older patients receiving chemotherapy for colorectal cancer: a randomised trial (GERICO). Br J Cancer 124:1949-1958, 2021
4. Mohile SG, Epstein RM, Hurria A, et al: Communication With Older Patients With Cancer Using Geriatric Assessment: A Cluster-Randomized Clinical Trial From the National Cancer Institute Community Oncology Research Program. JAMA Oncol 6:196-204, 2020
5. Puts M, Alqurini N, Strohschein F, et al: Impact of Geriatric Assessment and Management on Quality of Life, Unplanned Hospitalizations, Toxicity, and Survival for Older Adults With Cancer: The Randomized 5C Trial. J Clin Oncol 41:847-858, 2023
6. Soo WK, King MT, Pope A, et al: Integrated Geriatric Assessment and Treatment Effectiveness (INTEGERATE) in older people with cancer starting systemic anticancer treatment in Australia: a multicentre, open-label, randomised controlled trial. Lancet Healthy Longev 3:e617-e627, 2022
7. Paillaud E, Brugel L, Bertolus C, et al: Effectiveness of Geriatric Assessment-Driven Interventions on Survival and Functional and Nutritional Status in Older Patients with Head and Neck Cancer: A Randomized Controlled Trial (EGeSOR). Cancers (Basel) 14, 2022
8. Orum M, Eriksen SV, Gregersen M, et al: The impact of a tailored follow-up intervention on comprehensive geriatric assessment in older patients with cancer - a randomised controlled trial. J Geriatr Oncol 12:41-48, 2021
9. DuMontier C, Uno H, Hshieh T, et al: Randomized controlled trial of geriatric consultation versus standard care in older adults with hematologic malignancies. Haematologica 107:1172-1180, 2022
10. Loblaw DA, Prestrud AA, Somerfield MR, et al: American Society of Clinical Oncology Clinical Practice Guidelines: formal systematic review-based consensus methodology. J Clin Oncol 30:3136-40, 2012
11. The ADAPTE Collaboration: The ADAPTE Process: Resouce Toolkit for Guideline Adaptation. Version 2.0. Available from: http://www.g-i-n.net., 2009
