# Supplementary material for: Geriatric Assessment: ASCO Global Guideline
Source: JCO Glob Oncol. Author manuscript; Available in PMC 2026 Apr 1. (PMC13039670; doi:10.1200/GO-25-00276)
Supplement: Summary of Recommendations Table [file NIHMS2154859-supplement-Summary_of_Recommendations_Table.pdf]

ASCO® Guidelines

Geriatric Assessment: ASCO Global Guideline			
**Clinical Question**	**Recommendation**	**Evidence Quality**	**Strength**
What is the role of geriatric assessment in older adults with cancer to inform specific interventions to improve clinical outcomes in resource-constrained settings?	**1.1. Basic, Limited, Enhanced:** All patients with cancer aged 65 and over receiving systemic therapy and with geriatric assessment (GA)-identified impairments should have GA-guided management (GAM) included in their care plan. GAM includes using GA results to: 1) inform cancer treatment decision-making and 2) address impairments through appropriate interventions, counseling, telemedicine, and/or referrals.	M-H	S
For older patients who are considering undergoing antineoplastic therapy and other systemic treatments, which geriatric assessment tools and component elements should clinicians use to predict adverse outcomes (including antineoplastic therapy toxicity and mortality) and guide management in resource-constrained settings?	**2.1. Basic:** A geriatric evaluation should include at a minimum the use of a brief geriatric screening tool (G8 tool with a cutoff of ≤14 points is recommended).	M	S
	**2.1. Limited, Enhanced:** A GA should include high priority aging-related domains known to be associated with outcomes in older patients with cancer to include assessment of physical and cognitive function, emotional health, comorbid conditions, polypharmacy, nutrition, and social support.	H	S
	**2.2. Basic:** For patients who are identified as potentially vulnerable using a screening tool, the Panel recommends the Practical Geriatric Assessment (PGA) as one option for conducting a geriatric assessment. See the PGA tool and associated videos (How to Do A Geriatric Assessment, What to do with the Results of a Geriatric Assessment).	M	W
	**2.2. Limited and Enhanced:** The Panel recommends the Practical Geriatric Assessment (PGA) as one option for this purpose. See the PGA tool and associated videos (How to Do A Geriatric Assessment, What to do with the Results of a Geriatric Assessment).	M	W
	**2.3. All Levels:** Although the tools available in the PGA are available and validated in multiple languages, other similar tools may be more appropriate for some settings and languages, and those could be used if they include the relevant domains mentioned in Recommendation 2.1.	M	S
	**2.4. All Levels:** Cut-offs for GA-identified impairments have traditionally been developed in Maximal-resource settings of high-income countries. Therefore, differing cutoffs may be informed by locally validated research and practice.	M	W

www.asco.org/global-guidelines ©American Society of Clinical Oncology 2025. All rights reserved.
For licensing opportunities, contact licensing@asco.org

Geriatric Assessment: ASCO Global Guideline			
**Clinical Question**	**Recommendation**	**Evidence Quality**	**Strength**
What general (i.e., noncancer-specific) life expectancy data for community-dwelling patients should clinicians consider for estimating mortality and best inform treatment decision making for older patients with cancer in resource-constrained settings?	**3.1. All Levels:** In settings not adequately represented in the validated tools listed at ePrognosis,[a] clinicians may use actuarial life-expectancy tables, with preference given to those that consider quartiles of overall health status.	M	W
	**3.2. All Levels:** Based on the best clinical opinion of the Expert Panel, clinicians should use one of the validated tools listed at ePrognosis to estimate life expectancy greater than or equal to 4 years.<br>a. For settings outside the U.S. and Canada, the Expert Panel especially recommends the Suemoto Index. The most common variables considered in these indices include age, sex, comorbidities (e.g., diabetes, COPD), functional status (e.g., ADLs, IADLs, mobility), health behaviors and lifestyle factors (e.g., smoking status, body mass index), and self-rated health.<br>b. Several indices have "presence of cancer" as a relevant variable, answering "no" to this question will allow for non-cancer life expectancy, to consider competing risks of mortality.	M	S[b]<br>W[c]
How should geriatric assessment be used to guide management of older patients with cancer in resource-constrained settings?	**4.1. Basic:** Delphi consensus panels of experts have established approaches for implementing GA-guided care processes in older adults with cancer. The Expert Panel recommends that clinicians apply the results of GA to develop an integrated and individualized plan for patients that informs treatment selection by helping to estimate risks for adverse outcomes and to identify nononcologic problems that may be amenable to intervention. Based on clinical experience and the results of formal expert consensus studies, the Expert Panel suggests that clinicians consider G8 screening tool plus or minus GA results and when recommending treatment and that the information be provided to patients and caregivers to guide decision making for treatment. In addition, clinicians should implement targeted, GA-guided interventions to manage nononcologic problems (Table 2 in the full guideline).	M	W
	Qualifying statement for Recommendation 4.1. Basic: Most of these processes have been developed in Maximal-resource settings. Therefore, the implementation of GA-guided care may need to be adapted to the local context following the interventions listed in Table 2 (in the full guideline). A model of care utilizing the GA to inform clinicians to adapt treatment decisions for older patients with cancer may be reasonable in Basic settings.		

www.asco.org/global-guidelines ©American Society of Clinical Oncology 2025. All rights reserved.
For licensing opportunities, contact licensing@asco.org

Geriatric Assessment: ASCO Global Guideline			
**Clinical Question**	**Recommendation**	**Evidence Quality**	**Strength**
	**4.1. Limited:** Delphi consensus panels of experts have established approaches for implementing GA-guided care processes in older adults with cancer. The Expert Panel recommends that clinicians apply the results of GA to develop an integrated and individualized plan for patients that informs treatment selection by helping to estimate risks for adverse outcomes and to identify nononcologic problems that may be amenable to intervention. Based on clinical experience and the results of formal expert consensus studies, the Expert Panel suggests that clinicians consider GA results when recommending treatment and that the information be provided to patients and caregivers to guide decision making for treatment. In addition, clinicians should implement targeted, GA-guided interventions to manage nononcologic problems (Table 2 in the full guideline).	M	W
	Qualifying statement for Recommendation 4.1. Limited: Most of these processes have been developed in Maximal- or Enhanced-resource settings. Therefore, the implementation of GA-guided care may need to be adapted to the local context following the interventions listed in Table 2 (in the full guideline). A model of care which uses the GA to inform oncologists about adapting treatment decisions, along with appropriate referrals and co-management for older patients with cancer may be reasonable in Limited settings.		
	**4.1. Enhanced:** Delphi consensus panels of experts have established approaches for implementing GA-guided care processes in older adults with cancer. The Expert Panel recommends that clinicians apply the results of GA to develop an integrated and individualized plan for patients that informs treatment selection by helping to estimate risks for adverse outcomes and to identify nononcologic problems that may be amenable to intervention. Based on clinical experience and the results of formal expert consensus studies, the Expert Panel suggests that clinicians consider GA results when recommending treatment and that the information be provided to patients and caregivers to guide decision making for treatment. In addition, clinicians should implement targeted, GA-guided interventions to manage nononcologic problems (Table 2 in the full guideline).	M	W

Notes. The following recommendations (strong or conditional) and terminology [see guideline Data Supplement] represent reasonable options for patients depending on clinical circumstances and in the context of individual patient preferences. Recommended care should be accessible to patients whenever possible.

The strength of the recommendation is defined as follows, Strong: In recommendations for an intervention, the desirable effects of an intervention outweigh its undesirable effects. In recommendations against an intervention, the undesirable effects of an intervention outweigh its desirable effects. All or almost all informed people would make the recommended choice for or against an intervention.; Conditional/Weak: In recommendations for an intervention, the desirable effects probably outweigh the undesirable effects, but appreciable uncertainty exists. In recommendations against an intervention, the undesirable effects probably outweigh

www.asco.org/global-guidelines ©American Society of Clinical Oncology 2025. All rights reserved.
For licensing opportunities, contact licensing@asco.org

the desirable effects, but appreciable uncertainty exists. Most informed people would choose the recommended course of action, but a substantial number would not.

[a] University of California: ePrognosis, https://eprognosis.ucsf.edu
[b] Strong that it predicts mortality
[c] Weak that it improves outcomes or improves decision making

Abbreviations. ADLs, Activities of Daily Living; GA, geriatric assessment; GAM, GA-guided management; H, high; IADLs, Instrumental Activities of Daily Living; L, low; M, moderate; PGA, Practical Geriatric Assessment; S, strong; U.S., United States; W, weak

This summary table is derived from recommendations in *Geriatric Assessment: ASCO Global Guideline*. This is a tool based on an ASCO Guideline and is not intended to substitute for the independent professional judgment of the treating physician. Practice guidelines do not account for individual variation among patients. This tool does not purport to suggest any particular course of medical treatment. Use of the guideline and this tool are voluntary.

www.asco.org/global-guidelines ©American Society of Clinical Oncology 2025. All rights reserved.
For licensing opportunities, contact licensing@asco.org
